# Brief Digital Interventions to Support the Psychological Well-being of NHS Staff During the COVID-19 Pandemic: 3-Arm Pilot Randomized Controlled Trial

**DOI:** 10.2196/34002

**Published:** 2022-04-04

**Authors:** Johannes H De Kock, Helen Ann Latham, Richard G Cowden, Breda Cullen, Katia Narzisi, Shaun Jerdan, Sarah-Anne Munoz, Stephen J Leslie, Andreas Stamatis, Jude Eze

**Affiliations:** 1 Division of Rural Health and Wellbeing Institute of Health Research and Innovation School of Health, University of the Highlands and Islands Inverness United Kingdom; 2 Department of Clinical Psychology New Craigs Psychiatric Hospital Inverness United Kingdom; 3 Nairn Town and Country Hospital NHS Highland United Kingdom; 4 Human Flourishing Program Harvard University Cambridge, MA United States; 5 Institute of Health and Wellbeing University of Glasgow Glasgow United Kingdom; 6 Cardiac Unit Raigmore Hospital NHS Highland Inverness United Kingdom; 7 Exercise and Nutrition Sciences State University of New York Plattsburgh, NY United States; 8 Epidemiology Research Unit Department of Veterinary and Animal Science Northern Faculty, Scotland’s Rural College Inverness United Kingdom

**Keywords:** eHealth, public health, depression, anxiety, well-being, mobile health, intervention studies, staff, occupational health, NHS, intervention, support, COVID-19, randomized controlled trial

## Abstract

**Background:**

Health and social care staff are at high risk of experiencing adverse mental health (MH) outcomes during the COVID-19 pandemic. Hence, there is a need to prioritize and identify ways to effectively support their psychological well-being (PWB). Compared to traditional psychological interventions, digital psychological interventions are cost-effective treatment options that allow for large-scale dissemination and transcend social distancing, overcome rurality, and minimize clinician time.

**Objective:**

This study reports MH outcomes of a Consolidated Standards of Reporting Trials (CONSORT)-compliant parallel-arm pilot randomized controlled trial (RCT) examining the potential usefulness of an existing and a novel digital psychological intervention aimed at supporting psychological health among National Health Service (NHS) staff working through the COVID-19 pandemic.

**Methods:**

NHS Highland (NHSH) frontline staff volunteers (N=169) were randomly assigned to the newly developed NHSH Staff Wellbeing Project (NHSWBP), an established digital intervention (My Possible Self [MPS]), or a waitlist (WL) group for 4 weeks. Attempts were made to blind participants to which digital intervention they were allocated. The interventions were fully automated, without any human input or guidance. We measured 5 self-reported psychological outcomes over 3 time points: before (baseline), in the middle of (after 2 weeks), and after treatment (4 weeks). The primary outcomes were anxiety (7-item General Anxiety Disorder), depression (Patient Health Questionnaire), and mental well-being (Warwick-Edinburgh Mental Well-being Scale). The secondary outcomes included mental toughness (Mental Toughness Index) and gratitude (Gratitude Questionnaire-6).

**Results:**

Retention rates mid- and postintervention were 77% (n=130) and 63.3% (n=107), respectively. Postintervention, small differences were noted between the WL and the 2 treatment groups on anxiety (vs MPS: Cohen *d*=0.07, 95% CI –0.20 to 0.33; vs NHSWBP: Cohen *d*=0.06, 95% CI –0.19 to 0.31), depression (vs MPS: Cohen *d*=0.37, 95% CI 0.07-0.66; vs NHSWBP: Cohen *d*=0.18, 95% CI –0.11 to 0.46), and mental well-being (vs MPS: Cohen *d*=–0.04, 95% CI –0.62 to –0.08; vs NHSWBP: Cohen *d*=–0.15, 95% CI –0.41 to 0.10). A similar pattern of between-group differences was found for the secondary outcomes. The NHSWBP group generally had larger within-group effects than the other groups and displayed a greater rate of change compared to the other groups on all outcomes, except for gratitude, where the rate of change was greatest for the MPS group.

**Conclusions:**

Our analyses provided encouraging results for the use of brief digital psychological interventions in improving PWB among health and social care workers. Future multisite RCTs, with power to reliably detect differences, are needed to determine the efficacy of contextualized interventions relative to existing digital treatments.

**Trial Registration:**

ISRCTN Registry (ISRCTN) ISRCTN18107122; https://www.isrctn.com/ISRCTN18107122

## Introduction

### Background

Mental health (MH) has been deteriorating both globally and across the U.K. during the COVID-19 pandemic, with large-scale population studies reporting increased prevalence of depression and anxiety [1]. There are concerns that the public health crisis has disproportionately impacted the well-being of specialized populations, including health and social care workers (HSCWs) who provide valuable health care services. HSCWs exhibited high levels of preexisting MH problems before the COVID-19 pandemic [2-5], and recent evidence suggests that this group is at increased risk of experiencing worsening MH outcomes as a direct result of the COVID-19 pandemic [6-9]. MH problems in this population can affect morale and quality of care [5], which could have particularly devastating consequences for health systems because many parts of the world have been overwhelmed by the burden of COVID-19.

The majority of the general public [10] and health care staff [11] with common MH conditions do not access professional help, despite the existence of effective psychological treatments. Common reasons include a lack of service availability (especially in rural and remote areas), problems recognizing symptoms, treatment cost, and time constraints [11]. For HSCWs, the stigma surrounding mental illness and concerns about confidentiality have been identified as major barriers to accessing treatment and recovery, which can affect the quality of care HSCWs provide to patients [11,12]. Although research is ongoing, these barriers to treatment and downstream consequences for HSCWs, their families, and their patients appear to have been exacerbated by working through the COVID-19 pandemic [13].

Interventions designed to improve MH and psychological well-being (PWB) could help to mitigate the adverse effects of the COVID-19 pandemic on the well-being of HSCWs [8]. Digital psychological interventions overcome social distancing, rurality, and already overburdened clinician time constraints. Furthermore, digital interventions have a low cost relative to traditional psychological interventions, have already been widely used [14], are generally popular with users, and can be accessed anonymously at the user’s convenience. Evidence-based and rigorously tested digital interventions could allow for a rapid, economical, and large-scale dissemination of urgently needed psychological support for frontline staff working through the COVID-19 pandemic and its aftermath.

The past decade has seen digital psychological interventions being tested and validated in controlled and long-term follow-up studies, and the number of mobile MH interventions that are available is increasing rapidly. User reports indicate a significant increase in these apps downloaded during the first year of the COVID-19 pandemic in the U.K. [15] and in the U.S. [14]. Although validated digital interventions have been shown to be clinically efficacious, with effect sizes similar to that of traditional or face-to-face therapy [16], there is little research into the efficacy of such treatment approaches for frontline HSCWs who have been working through the COVID-19 pandemic [17]. Furthermore, the majority of digital psychological interventions during the public health crisis have been focused on decreasing symptoms associated with psychopathology (ie, depression and anxiety); few have been designed with end-user input (patient and public involvement [PPI]) and oriented toward enhancing PWB [17]. Given the unprecedented scale of the COVID-19 pandemic’s burden on HSCWs, specialized and contextual interventions are needed to support the MH of this population [18].

### This Study

This study aims to provide preliminary evidence on the use of digital psychological interventions to support frontline staff psychological health in the context of the COVID-19 pandemic. In a pilot randomized controlled trial (RCT), we evaluated the use of 2 smartphone apps designed to support PWB against a control condition (waitlist [WL]): (1) My Possible Self (MPS) [19], which is a well-established validated app with a track record of showing significant improvements in depression, anxiety, and stress in users over a short period [19], together with good user satisfaction rates; (2) the National Health Service (NHS) Highland Wellbeing Project (NHSWBP), which is a PPI-informed, brief, fully automated, and context- (COVID-19 pandemic) and population-specific (frontline staff) digital psychological intervention built on the MPS model and wireframe to promote PWB among HSCWs.

We predicted that symptoms of depression and anxiety would decline among users randomly allocated to receive digital psychological interventions, while mental well-being would increase, relative to the WL group. Two positive psychology concepts shown to mitigate the negative effects of depression and anxiety and promote positive adaptation in the face of adversity (eg, what frontline staff are facing while working through a pandemic) that are amenable to change are mental toughness (MT) [20] and gratitude [21]. We also predicted that use of digital psychological interventions would increase MT and gratitude. Although we predicted both digital interventions to yield improvements relative to the WL group, we expected that the NHSWBP group would show greater rates of improvements because it is designed specifically for the COVID-19 context. To the best of our knowledge, this is the first trial to examine fully automated, brief digital psychological interventions aimed to support the psychological health of frontline staff working through the COVID-19 pandemic.

## Methods

### Eligibility Criteria

Participants were required to meet the following criteria: UK resident, aged 18 years and over, working in the NHSH as a health or social care worker during the COVID-19 pandemic, and owning an internet-enabled mobile phone. Both clinical (doctors, nurses, allied health professionals) and nonclinical (eg, administrators) staff were eligible.

### Sample, Setting, and Procedure

Given that this was a pilot trial being conducted in a limited time, the sample size targets were based on pragmatic factors rather than an expectation of having the power to enable detection of the expected effect sizes. Participants were recruited locally and online between July and September 2020. Data collection took place at the beginning, middle, and end of the pilot RCT intervention phase, which ran from September to October 2020. Recruitment was conducted digitally by NHSH human resources, which included emails and electronic newsletters. Further recruitment was conducted via general physician (GP) practice managers, as well as heads of departments in primary and secondary care. A secondary level of recruitment was conducted on social media; a page for the study was created on Twitter, Facebook, and LinkedIn. Paid advertisements were also used on Facebook and LinkedIn to promote the study. Across all recruitment routes, interested individuals were directed to a secure data collection website via a weblink, where they first reviewed information about the study and provided electronic consent to participate. Eligible participants then completed a baseline survey, after which they were randomized to a condition. All participants were asked to complete follow-up surveys after the first 2 weeks of the intervention (middle) and 4 weeks after baseline following completion of the intervention period. At each assessment point, participants accessed the survey via a weblink sent to them in an email message. Demographic and basic clinical information was collected during the baseline survey, which included age, gender, place of work, job type, level of education, years of experience, previous psychiatric diagnosis, and whether the person was working directly with COVID-19 patients.

This study was part of the Scottish Government’s Rapid Research into COVID-19, and time restrictions limited recruitment activities; it was not possible to extend recruitment activities or product development beyond the grant’s funding time frame. Written informed consent was provided by all participants. The RCT was approved by the NHS Health Research Authority (20/SW/0098) and registered at the ISRCTN Registry (ISRCTN18107122). The intervention phase ran from September 7 to October 5, 2020, during the start of the second wave of the COVID-19 pandemic in Scotland.

### Design

A mixed factorial repeated measures design was used, with full randomization to 3 parallel groups.

### Randomization

A research assistant not involved in the RCT randomized participants after baseline using computerized simple randomization. Allocation was either to the MPS, NHSWBP, or WL group. Participants received advice of their group assignment by email. Participants were blinded to which intervention they received by styling the 2 interventions and communications to participants similarly. Participants downloaded the same app from the iTunes/App Store/Play Store, and a code was sent back to them to initiate the intervention that they received.

### Interventions

#### My Possible Self

MPS (version 2.0.0) is a tried and tested, NHS-approved [22] smartphone well-being app with a validated track record of showing significant improvements in depression, anxiety, and stress in its users over a short period [19]. It is fully automated and freely available to NHS staff. This intervention has modules that cover a variety of topics and can be accessed in any order, including coping effectively with depression and anxiety, enhancing happiness, improving sleep quality, and practicing mindfulness.

#### NHS Highland Staff Wellbeing Project

The NHSWBP is a PPI-informed, brief, fully automated, and context- (COVID-19) and population-specific (NHSH frontline staff) digital psychological intervention (smartphone app) based on the MPS. It utilizes the tried and tested cognitive behavioral therapeutic (CBT) and positive psychological techniques delivered via the MPS [19] smartphone app’s modules. There were a number of ways in which the NHSWBP app differed from the MPS app. First, the NHSWBP was presented as a coherent narrative with a fictional character, a Scottish nurse named Iona, who guided participants through the linear narrative of the app and its interventions. Participants also received automated text messages from Iona to engage them in the overall narrative and to motivate continued engagement with the intervention. Second, the NHSWBP was designed following PPI feedback, which included input about which MPS modules were most relevant, the duration of the modules, and the coherence and flow of the presentation format. Third, the NHSWBP provided links to local and national 24-hour support services. Similarly to the MPS, participants were able to monitor and record their mood and levels of distress or well-being, add notes, and identify and record triggers for low mood and anxiety. The intervention lasted for 4 weeks and consisted of 2 parts: part 1 (duration 2 weeks) focused on increasing participants’ happiness, resilience, and well-being, and part 2 (duration 2 weeks) focused on managing low mood and anxiety effectively. The NHSWBP was codesigned by the University of the Highlands and Islands (UHI), the NHSH, and the software and technical team that supports the MPS app. The NHSWBP was designed using the MPS app platform and participant communication system, owing to its established track record and NHS approval.

### Primary Outcomes

Postintervention was the primary timepoint for all outcomes.

#### Depression

The Patient Health Questionnaire (PHQ-9) [23] was used to measure depression. The 9 items ask participants to consider how bothered they have been over the past 2 weeks according to each statement (eg, “feeling tired or having little energy”). The questionnaire score ranges from 0 to 27; each question is given a 4-point response (0=not at all to 3=nearly every day). The questionnaire has demonstrated diagnostic validity [23]. This measure has been used extensively in the U.K. [24] and internationally [25] to measure levels of depression in various population settings during the COVID-19 pandemic.

#### Anxiety

The 7-item General Anxiety Disorder (GAD-7) [26] scale was used to measure anxiety. Similar to the PHQ-9, each item asks the respondent to consider the statement based on how much they have been bothered over a 2-week period (eg, “feeling nervous anxious or on edge”). Each item is scaled from 0 (not at all) to 3 (nearly every day), with a total score range of 0-21. A number of studies during the COVID-19 pandemic have used the GAD-7 to measure levels of anxiety in various UK and international population settings, including in frontline staff working through this pandemic [8,24].

#### Mental Well-being

Mental well-being was measured using the Warwick-Edinburgh Mental Well-being Scale (WEMWBS) [27]. The scale consists of 14 items used to measure subjective well-being and psychological functioning. The wording of each item is positive and aimed to address positive aspects of MH. Responses are completed using a 5-point scale (1=none of the time to 5=all of the time); the total score ranges from 14 to 70. The WEMWBS has been validated for use in the U.K. [27] and has been used internationally [28] and in the U.K. [29] to measure the MWB of HSCWs during this pandemic.

### Secondary Outcomes

#### Mental Toughness

The Mental Toughness Index (MTI) [20] was used to measure MT. The 8 items (eg, “I can find a positive in most situations”) are rated using a 7-point response format (1=false, 100% of the time, to 7=true, 100% of the time), with responses combined for a total MT score. Studies involving samples from different countries (eg, Australia, South Africa) [20,30-32] have adduced evidence that supports the construct validity (eg, convergent, criterion) of the MTI. In prior studies, internal consistency reliability estimates for the MTI have been ≥0.87 [30,31,33].

#### Gratitude

Participants completed the Gratitude Questionnaire-6 (GQ-6) [34], which is a 6-item measure of dispositional gratitude. Items (eg, “I have so much in life to be thankful for”) are rated on a 7-point response format (1=strongly disagree to 7=strongly agree), 2 of which are reverse-scored. Evidence from studies involving diverse samples [34-36] supports the factorial validity of the GQ-6 as a measure of the grateful disposition that is conceptually distinct from related constructs (eg, hope, optimism). Internal consistency reliability values reported in previous research have been ≥0.82 [34-36].

### Statistical Analyses

Statistical analyses and data manipulations were implemented using R (R Core Team) [37]. Baseline characteristics of participants randomly allocated to the 3 intervention groups were compared using the chi-square test. The effects of the MPS and NHSWBP interventions on psychological measures were examined using intention-to-treat (ITT) analyses that included data from all participants who completed the baseline assessment and any follow-up assessment. No imputation was used for missing data. Standard regression models assume independent observations. To adequately account for the dependencies in the data, we adopted the linear mixed modeling (LMM) approach [38] for the analyses of the data. This approach is appropriate for studying the relationships and sources of variation in the data set. It uses all available data and efficiently handles missing data, thereby avoiding listwise deletion. LMM models all sources of variation in the data and avoids the need for data imputation. Each psychological outcome was modeled as a function of time, treatment group, and their interaction and adjusting for random effects due to individual differences and repeated observations from each participant. The models allow for each participant to have a different trajectory. Model parameters were estimated using the restricted maximum likelihood. The best model was selected using the likelihood ratio test. Based on the chosen model, marginal means were estimated and multiple comparisons of groups by time interaction tests conducted using sets of Tukey-adjusted interaction contrasts [39]; degrees of freedom were calculated using the Kenward-Roger test [40].

The effects were tested at a significance level of .05, adjusted depending on the number of contrasts in multiple tests. Cohen *d* was calculated by standardizing the mean difference of within and between groups using the square root of the sum of all the variance components from the mixed models. This is to adequately represent the study design and account for all sources of variation in data [41,42].

Linear regression slopes of each psychological measure were modeled as a function of time, treatment, and time-treatment interaction. Pairs of the slopes were then compared using the lsmean approach of Lenth (2016) to determine the intervention that brought about a higher rate of change in the mean of the psychological measures [43]. This analysis used data for the 3 time periods and modeled the average trend for each of the measured outcomes. A second analysis adjusted for the baseline by entering the baseline values of the outcome of interest as a covariate in the mixed effect model that also included group-time intervention as a fixed effect.

## Results

### Randomization and Study Attrition

**Figure 1:**
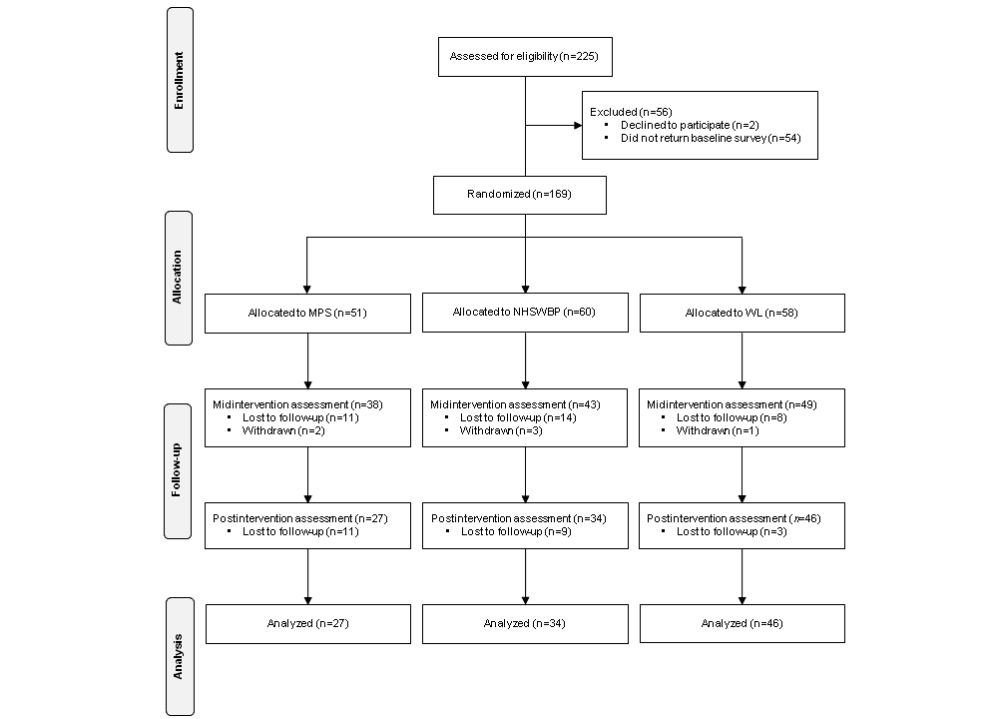
Details of enrolment into the trial.

Details of enrolment into the trial, organized according to the Consolidated Standards of Reporting Trials (CONSORT) guidelines [44], are shown in Figure 1. Of the 225 people who expressed an interest in the study, completed eligibility screening information, and provided consent to participate, 54 (24%) did not complete the baseline questionnaire and 2 declined to participate (0.9%). These 56 individuals were excluded from the analyses, leaving a study sample of 169 (75.1%) participants. The distribution of participant characteristics at baseline and postintervention is reported in Table 1. Participants were mostly female (n=149, 88.2%) and nurses (n=48, 28.4%), doctors (n=39, 23.1%), allied health professionals (n=21, 12.4%), administrative staff (n=16, 9.5%), health care assistants (n=8, 4.7%), carers (n=6, 3.6%), and other HSWCs (n=31, 18.3%). At baseline, 53 (31.4%) of the 169 participants met the criteria for low mental well-being (WEMWBS score<40), 51 (30.2%) met the criteria for possible depression (PHQ-9>10), and 46 (27.2%) met the criteria for possible anxiety (GAD-7≥10).

The 3 groups did not differ on the professional, demographic, and clinical history variables assessed at baseline (*P*>.05). The rate of attrition for the total sample was 23.0% in the middle of the intervention and 36.7% at postintervention assessment. Rates of attrition across the demographic, professional, and clinical characteristics of the participants are presented in Table 1. Participants who worked fewer hours per week and were not employed in administrative positions (eg, doctors) were more likely to drop out during the intervention (all *P*≤.04). Postintervention, attrition in the MPS (47.1%) and NHSWBP (43.3%) groups was higher (*χ*^2^_2_=9.89, *P*=.01) compared to the WL group (20.7%). In all 3 treatment conditions, there was little evidence of baseline differences on the demographic, employment, and clinical history variables between participants who were retained and those who dropped out of the study (all *P*>.05).

**Table 1:** Distribution of participant characteristics at baseline and postintervention.

Characteristic		Baseline (N=169), n (%)	Postintervention (N=107), n (%)	Attrition rate
**Gender**				
	Female	149 (88.2)	94 (87.9)	0.37
	Male	20 (11.8)	13 (12.1)	0.35
**Age (years)**				
	18-25	4 (2.4)	4 (3.7)	N/A^a^
	26-30	10 (5.9)	4 (3.7)	0.60
	31-40	31 (18.3)	16 (15.0)	0.48
	>40	124 (73.4)	83 (77.6)	0.33
**Education level**				
	Undergraduate or lower	65 (38.5)	40 (37.4)	0.38
	Postgraduate or higher	104 (61.5)	67 (62.6)	0.36
**Type of employment**				
	Nurse	48 (28.4)	30 (28.0)	0.38
	Doctor	39 (23.1)	21 (19.6)	0.46
	Allied health professional	21 (12.4)	12 (11.2)	0.43
	Administrative	16 (9.5)	13 (12.1)	0.19
	Carer	6 (3.6)	4 (3.7)	0.33
	Health care assistant	8 (4.7)	4 (3.7)	0.50
	Other	31 (18.3)	23 (21.5)	0.26
**Years of employment experience**				
	<2	14 (8.3)	11 (10.5)	0.21
	2-5	13 (7.8)	8 (7.6)	0.38
	5-10	21 (12.6)	10 (9.5)	0.52
	>10	119 (71.3)	76 (72.4)	0.36
**Workplace**				
	Community, GP^b^, and PC^c^	73 (43.7)	41 (39.0)	0.44
	Hospital	74 (44.3)	51 (48.6)	0.31
	Other	20 (12.0)	14 (13.3)	0.30
	N/A	N/A	1 (1.0)	N/A
**Hours worked/week**				
	<20	8 (4.7)	3 (2.8)	0.62
	20-30	31 (18.3)	18 (16.8)	0.42
	30-40	100 (59.2)	61 (57.0)	0.39
	>40	30 (17.8)	25 (23.4)	0.17
**Work with COVID-19 patients**				
	No	129 (77.2)	84 (80.0)	0.35
	Yes	38 (22.8)	21 (20.0)	0.45
**Level of disruption**				
	No disruption	3 (1.8)	1 (0.9)	0.67
	Minor	15 (8.9)	10 (9.3)	0.33
	Moderate	65 (38.0)	41 (38.3)	0.37
	Major	66 (39.0)	40 (37.4)	0.39
	Severe	20 (12.0)	15 (14.0)	0.25
**Shielding**				
	No	145 (85.8)	93 (86.9)	0.36
	Yes	7 (4.1)	5 (4.7)	0.29
	Family member is shielding	17 (10.1)	9 (8.4)	0.47
**Psychiatric disorder**				
	No	131 (77.5)	78 (72.9)	0.40
	Yes	38 (22.5)	29 (27.1)	0.23

^a^N/A: not applicable.

^b^GP: general physician.

^c^PC: primary care.

### Outcomes

**Figure 2:**
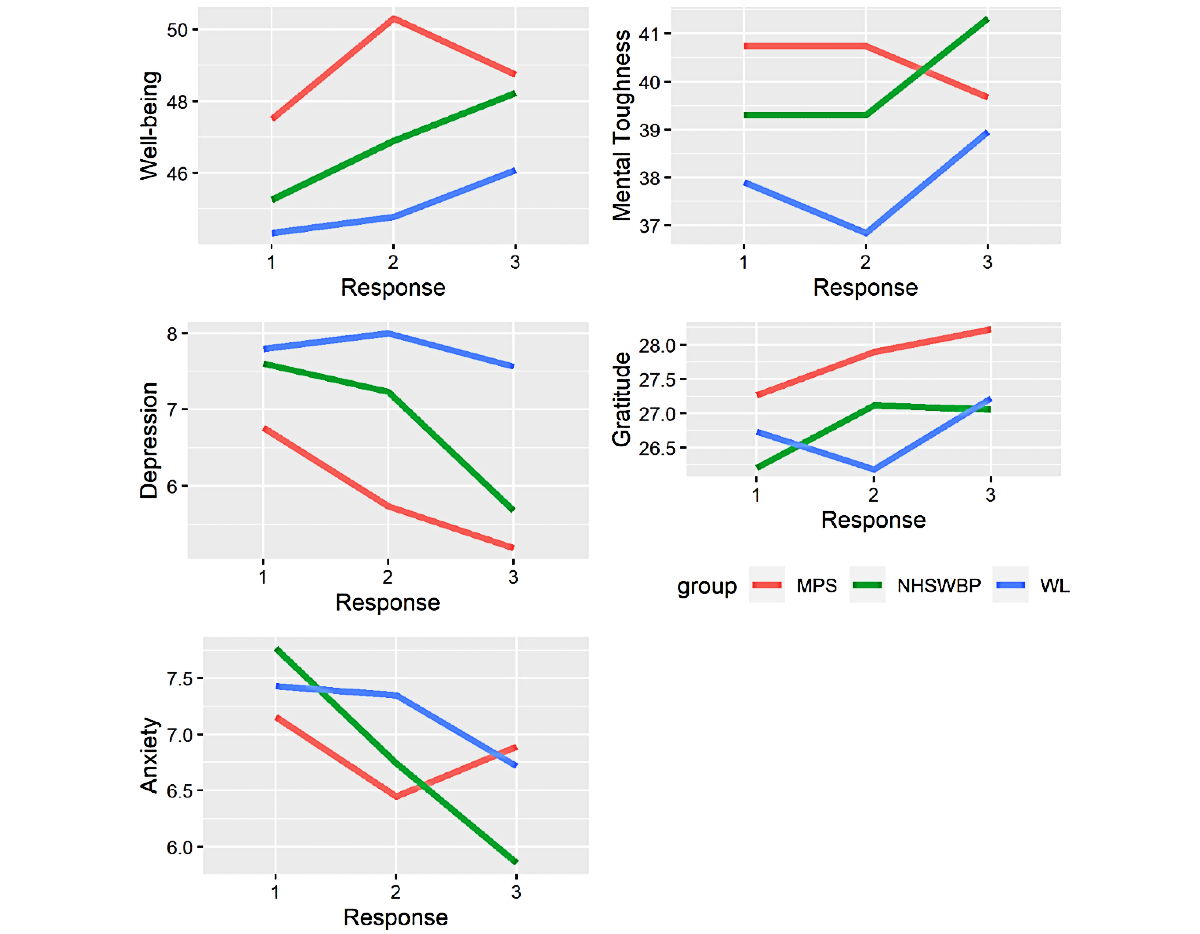
Effect size plot for the 3 conditions on the primary and secondary outcome measures at baseline, midintervention, and postintervention. MPS: My Possible Self; NHSWBP: National Health Service Highland Staff Wellbeing Project; WL: waitlist.

Table 2 reports the observed mean scores for each outcome at baseline, midintervention, and postintervention in the 3 treatment groups. Figure 2 depicts these scores for the 3 groups on the primary and secondary outcome measures at baseline, midintervention, and postintervention.

**Table 2:** Descriptive statistics for outcomes at baseline, midintervention, and postintervention in each treatment condition.

Outcome		MPS^a^, mean (SD)	NHSWBP^b^, mean (SD)	WL^c^, mean (SD)
**Anxiety**				
	Baseline	7.16 (5.60)	7.77 (4.87)	7.43 (5.10)
	Midintervention	6.45 (5.03)	6.74 (4.69)	7.35 (5.23)
	Postintervention	6.89 (5.71)	5.85 (3.66)	6.72 (5.59)
**Depression**				
	Baseline	6.76 (5.04)	7.60 (4.31)	7.80 (5.23)
	Midintervention	5.74 (4.31)	7.23 (5.47)	8.00 (5.06)
	Postintervention	5.18 (3.27)	5.68 (4.39)	7.56 (6.26)
**Mental well-being**				
	Baseline	47.5 (10.2)	45.3 (8.65)	44.3 (10.1)
	Midintervention	50.3 (9.75)	46.9 (8.68)	44.8 (10.4)
	Postintervention	48.7 (10.1)	48.2 (7.38)	46.1 (11.1)
**MT^d^**				
	Baseline	40.7 (8.04)	39.3 (6.84)	37.9 (9.81)
	Midintervention	40.7 (9.10)	39.3 (9.55)	36.8 (9.20)
	Postintervention	39.7 (9.80)	41.3 (8.33)	39.0 (10.5)
**Gratitude**				
	Baseline	27.3 (3.46)	26.2 (3.35)	26.7 (3.73)
	Midintervention	27.9 (3.63)	27.1 (4.14)	26.2 (4.30)
	Postintervention	28.2 (4.23)	27.1 (4.24)	27.2 (3.72)

^a^MPS: My Possible Self.

^b^NHSWBP: National Health Service Highland Staff Wellbeing Project.

^c^WL: waitlist.

^d^MT: mental toughness.

Mean scores across all outcomes indicated higher levels of functioning in the MPS group compared to the NHSWBP and WL groups at baseline. The NHSWBP group saw the largest increase in mental well-being scores across groups between baseline (mean 45.3 [SD 8.65]) and postintervention (mean 48.2 [SD 7.38]).

Levels of depression decreased from baseline to midintervention for both MPS and NHSWBP groups, while the WL group in contrast saw a rise in depression scores from baseline (mean 7.80 [SD 5.23]) to midintervention (mean 8.00 [SD 5.06]). Mean levels of depression for the MPS and NHSWBP groups continued to decrease over time, with the NHSWBP group showing the largest decrease from 7.60 (SD 4.31) at baseline to 5.68 (SD 4.39) postintervention. The WL group showed a slight decrease in levels of depression at the postintervention measurement (mean 7.56 [SD 6.26]).

Levels of anxiety decreased across all groups from baseline to postintervention. The WL group showed a consistent decrease in anxiety levels from baseline (mean 7.43 [SD 5.10]) to midintervention (mean 7.35 [SD 5.23]) to postintervention (mean 6.72 [SD 5.59]). The MPS group indicated a decrease in levels of anxiety from baseline (mean 7.16 [SD 5.60]) to midintervention (mean 6.45 [SD 5.03]) and a slight increase postintervention when compared to midintervention (mean 6.89 [SD 5.71]). Baseline levels of anxiety were highest in the NHSWBP group (mean 7.77 [SD 4.87]), and this group also evidenced the greatest decrease in anxiety levels postintervention (mean 5.68 [SD 4.39]).

Levels of gratitude in the MPS group increased from baseline to midintervention to postintervention. Mean gratitude scores in the NHSWBP group increased from baseline to midintervention and then remained constant postintervention. The WL group saw a slight decrease in levels of gratitude from baseline (mean 26.7 [SD 3.73]) to midintervention (mean 26.2 [SD 4.3]), with an increase noted postintervention (mean 27.2 [SD 3.72]).

Levels of MT in the NHSWBP group remained constant from baseline (mean 39.3 [SD 6.84]) to midintervention (mean 39.3 [SD 9.55]), before increasing slightly postintervention to 41.3 (SD 8.33). For the MPS group, these levels also remained constant from baseline (mean 40.7 [SD 8.04]) to midintervention (mean 40.7 [SD 9.10]), before decreasing postintervention (mean 39.7 [SD 9.80]). For the WL group, MT levels decreased from baseline (mean 37.9 [SD 9.81]) to midintervention (mean 36.8 [SD 9.20]), before increasing to 39.0 (SD 10.5) postintervention.

### Standardized Effect Size

The between- and within-group effect sizes (standardized mean difference) on the primary and secondary outcomes calculated using observed means are presented in Tables 3 and 4. Postintervention, between-group effect sizes were small to medium for the primary (NHSWBP vs MPS Cohen *d*=0.19 to –0.20; WL vs MPS Cohen *d*=–0.04 to 0.36; WL vs NHSWBP Cohen *d*=0.06 to –0.18) and secondary outcome measures. The results showed a consistent pattern of greater improvements in depression, anxiety, well-being, MT, and gratitude among participants in the digital intervention groups (MPS and NHSWBP) postintervention compared to the WL group.

Postintervention, a small difference was noted between the WL and the 2 treatment groups on anxiety (vs MPS: Cohen *d*=0.07, 95% CI –0.20 to 0.33; vs NHSWBP: Cohen *d*=0.06, 95% CI –0.19 to 0.31), depression (vs MPS: Cohen *d*=0.37, 95% CI 0.07-0.66; vs NHSWBP: Cohen *d*=0.18, 95% CI –0.11 to 0.46), and mental well-being (vs MPS: Cohen *d*=–0.04, 95% CI –0.62 to –0.08; vs NHSWBP: Cohen *d*=–0.15, 95% CI –0.41 to 0.10). The NHSWBP group generally had larger within-group effects than the other groups. Within-group effects for both MPS and NHSWBP groups ranged from small to medium based on observed means (MPS Cohen *d*=–0.31 to 0.25, NHSWBP Cohen *d*=–0.38 to 0.24). For the WL group, within-group effects were generally small for the primary outcomes (Cohen *d*=–0.12 to 0.16) and small to medium for the secondary outcome measures (Cohen *d*=0.13-0.27).

**Table 3:** Between-group effects calculated using observed means.

Between-group effects	Anxiety, Cohen *d* (95% CI)	Depression, Cohen *d* (95% CI)	Mental well-being, Cohen *d* (95% CI)	MT^a^, Cohen *d* (95% CI)	Gratitude, Cohen *d* (95% CI)
NHSWBP^b^ vs MPS^c^	0.01 (–0.26 to 0.28)	0.19 (–0.12 to 0.50)	–0.20 (–0.48 to 0.08)	–0.07 (–0.41 to 0.27)	–0.26 (–0.57 to 0.06)
WL^d^ vs MPS	0.07 (–0.20 to 0.33)	0.37 (0.07-0.66)	–0.04 (–0.62 to –0.08)	–0.31 (–0.64 to 0.02)	–0.28 (–0.58 to 0.02)
WL vs NHSWBP	0.06 (–0.19 to 0.31)	0.18 (–0.11 to 0.46)	–0.15 (–0.41 to 0.10)	–0.24 (–0.55 to 0.07)	–0.02(–0.32 to 0.27)

^a^MT: mental toughness.

^b^NHSWBP: National Health Service Highland Staff Wellbeing Project.

^c^MPS: My Possible Self.

^d^WL: waitlist.

**Table 4:** Within-group effects calculated using observed means.

Within-groups effects			Anxiety, Cohen *d* (95% CI)		Depression, Cohen *d* (95% CI)		Mental well-being, Cohen *d* (95% CI)	MT^a^, Cohen *d* (95% CI)	Gratitude, Cohen *d* (95% CI)
**MPS^b^**									
	Postintervention vs. baseline	–0.05 (–0.72 to 0.63)		–0.31 (–1.08 to 0.46)		0.11 (–0.58 to 0.80)		0.13 (–0.97 to 0.72)	0.25 (–0.54 to 1.04)
	Postintervention vs. midintervention	0.07 (–0.60 to 0.75)		–0.11 (–0.87 to 0.66)		–0.14 (–0.83 to 0.55)		0.13 (–0.97 to 0.72)	0.08 (–0.70 to 0.87)
**NHSWBP^c^**									
	Postintervention vs. baseline	–0.32 (–0.94 to 0.29)		–0.38 (–1.08 to .32)		0.27 (–0.36 to 0.90)		0.24 (–0.53 to 1.01)	0.22 (–0.50 to 0.94)
	Postintervention vs. midintervention	–0.15 (–0.77 to 0.47)		–0.30 (–1.00 to 0.39)		0.12 (–0.51 to 0.75)		0.24 (–0.53 to 1.01)	–0.01 (–0.73 to 0.70)
**WL^d^**									
	Postintervention vs. baseline	–0.12 (–0.67 to 0.43)		–0.05 (–0.67 to 0.58)		0.16 (–0.40 to 0.72)		0.13 (–0.56 to 0.81)	0.13 (–0.52 to 0.77)
	Postintervention vs. midintervention	–0.11 (–0.66 to 0.45)		–0.09 (–0.71 to 0.54)		0.12 (–0.44 to 0.68)		0.25 (–0.44 to 0.94)	0.27 (–0.37 to 0.91)

^a^MT: mental toughness.

^b^MPS: My Possible Self.

^c^NHSWBP: National Health Service Highland Staff Wellbeing Project.

^d^WL: waitlist.

### Comparing the Rate of Change per Condition

Table 5 shows the rate of change observed due to the interventions by comparing the trends in the effect size plot. When the gradient of the slopes of linear regression of each psychological outcome was estimated as a function of time, treatment, and time-treatment interaction, each group demonstrated improvements in average scores on all the 3 outcomes over the study period. Although the test for differences between the slopes of each group did not reach statistical significance (*P*>.05), the rate of improvement in anxiety, depression, and mental well-being was largest among those in the NHSWBP group. The WL group evidenced the smallest rate of change on each of the 3 outcomes.

For the secondary outcome measures, the average scores for MT increased in the NHSWBP and WL groups, whereas a slight decline was found in the MPS group. The rate of increase in MT was greatest in the NHSWBP group. We also observed the average scores for gratitude to increase for all 3 groups, with the greatest rate of increase in the MPS group. The smallest rate of increase in gratitude was found in the WL group.

**Table 5:** Response trends comparing baseline to postintervention in each treatment condition.

Outcome		Effect estimate (SE)	95% CI
**Anxiety**			
	MPS^a^	–0.05 (0.66)	–1.35 to 1.26
	NHSWBP^b^	–0.79 (0.61)	–1.98 to 0.41
	WL^c^	–0.35 (0.55)	–1.42 to 0.72
**Depression**			
	MPS	–0.76 (0.62)	–1.99 to 0.48
	NHSWBP	–0.94 (0.57)	–2.06 to 0.18
	WL	–0.18 (0.51)	–1.19 to 0.83
**Mental well-being**			
	MPS	1.11 (1.22)	–1.29 to 3.50
	NHSWBP	1.62 (1.11)	–0.57 to 3.81
	WL	0.88 (0.99)	–1.07 to 2.83
**MT^d^**			
	MPS	–0.54 (1.15)	–2.80 to 1.71
	NHSWBP	0.97 (1.04)	–1.08 to 3.02
	WL	0.52 (0.94)	–1.33 to 2.37
**Gratitude**			
	MPS	0.59 (0.50)	–0.39 to 1.57
	NHSWBP	0.39 (0.45)	–0.49 to 1.28
	WL	0.23 (0.40)	–0.56 to 0.84

^a^MPS: My Possible Self.

^b^NHSWBP: National Health Service Highland Staff Wellbeing Project.

^c^WL: waitlist.

^d^MT: mental toughness.

### Program Adherence

Adherence, defined as the extent to which participants engaged with the intervention, was examined for both the NHSWBP and MPS groups with respect to average interactions per user. Adherence was deemed to be good for both digital interventions, with participants in the NHSWBP group interacting, on average, 37.4 times with the intervention (more than once per day, on average) during the month-long intervention, while those in the MPS group interacted, on average, 37.5 times. None of the adherence indices correlated with demographic, clinical history, and primary and secondary outcome data obtained at baseline. No harmful or unintended effects were reported by the participants.

### Post hoc Power Calculation

Instead of using the observed effect size to calculate the post hoc study power (which could introduce bias) [45], we used the observed sample size and a fixed threshold for power and significance and calculated the smallest effect size that could be reliably detected with our sample size. By using this approach, together with our study design, we found that our study could detect an effect size of at least f=0.27 at 80% power.

## Discussion

### Principal Findings

We conducted a novel pilot RCT to evaluate 2 brief, fully automated digital health interventions in a sample of frontline staff working through the second wave of the COVID-19 pandemic. The trial proceeded successfully during challenging circumstances in the shadow of the second wave of the COVID-19 pandemic in the U.K. Our low-cost study demonstrated that it was possible to recruit 169 people working in a small NHS board within a short duration and deliver a technically innovative intervention on a modest financial budget. The NHSWBP app was designed with end-user (PPI) input and worked well throughout, with good adherence and no major flaws or bugs, nor evidence of harm reported by the participants. Furthermore, the WL control design was effective at retaining participants (who otherwise might have lost interest in the study and dropped out if it was just a no-treatment control rather than a WL). We also accumulated rich background data that could assist in identifying the possible drivers of dropout, which could be used to modify the design of the intervention to improve retention in a larger future trial. Although this was a pilot trial that we conducted during a prescribed limited time with a relatively small sample size, the findings of this study provide encouraging results for future full trials of digital psychological interventions that are designed to support the MH and PWB of HSCWs who are working under conditions of extreme stress.

There are 3 key findings of interest in this study. First, the primary outcomes investigated showed decreases in levels of depression (NHSWBP and MPS groups) and anxiety (NHSWBP group) when compared to the WL group. The rate of decrease in depression and anxiety symptoms was the greatest among those exposed to the NHSWBP intervention. Our results also indicate that the individuals exposed to the digital interventions and WL conditions experienced an increase in mental well-being, with the rate of increase again shown to be the greatest for those exposed to the NHSWBP intervention.

Second, for the secondary outcomes investigated, our results showed increases in MT for the NHSWBP and WL groups, with the rate of increase in MT again being the greatest for those exposed to the NHSWPB intervention. All groups experienced an increase gratitude over the treatment period, with the rate of change being the greatest for those exposed to the MPS intervention. Overall, our results show greater rates of symptom improvements in the digital intervention groups than in the WL condition. Concerning gratitude, our efforts add on the new research direction that investigates how it can be enhanced to be favorable to MH outcomes [46]. With regard to MT, our study adds to a growing field suggesting that MT could buffer the negative effects of depression and anxiety and promote adaptive MH outcomes [47]. Our research adds an interventional design and avoids the commonly used cross-sectional designs used to investigate gratitude [46] and MT [48]. Our pilot intervention showed promise in terms of both the traditional view of clinical psychology (ie, anxiety, depression) and also with regard to the science of positive psychology and character strengths [49].

Third, our results also provide preliminary support for the development or modification of digital interventions to be context specific, as of the 2 interventions tested, the NHSWBP showed greater rates of symptom improvement. Future trials assessing context-specific digital interventions for specialized populations in larger samples are warranted, as there is good reason to believe those larger studies will demonstrate efficacy. The digital nature of these interventions was seen to be safe, cost effective, and rapidly modifiable to context. The future application of similar, context-specific, robustly tested interventions could be scalable to other contexts with MH human resource needs [50].

### Limitations and Future Research Directions

There are several limitations of this study that need to be acknowledged. First, participants included a small sample of HSCWs from a single NHS site. Although the majority of respondents were female, this does not differ dramatically from the gender composition of the whole HSCW workforce in NHS Highland [51]. As our objective was to gather preliminary evidence on the potential benefits of 2 digital interventions in this population, the study was not powered as an efficacy trial, and so CIs around estimated effects were wide (indicating the small sample may have contributed to statistical uncertainty) and the findings may not be generalizable to other populations and HSCWs living in other contexts. Second, the treatment period was restricted to 4 weeks, and it is possible that changes in MH and PWB require more engagement in the intervention materials. In addition, some outcomes may change more gradually and require a longer period to improve. For example, gratitude exercises can orientate a person to experience more grateful emotions, but it could take more than 4 weeks for changes in dispositional gratitude to emerge. Future research would do well to track and monitor whether gains that are made during treatment are maintained or change over time. Third, the MPS app was publicly available for download throughout the duration of our study, and participants were not restricted to use other modalities or medications during this pilot, which raises the possibility that treatment effects might be cross-contaminated. Fourth, the attrition rate postintervention was 36.7%. The dropout rate was lowest in the WL group, which is likely attributable to participants waiting to receive either of the digital interventions. Although we did not find any substantial evidence of attrition bias, it is possible that participants who dropped out from the intervention groups were less satisfied with the program or experienced less than positive outcomes. Additional research is needed to explore the mechanisms underlying the effects that emerged in this study and to identify the relative contributions of the components that constituted each of the digital interventions. There may also be value in taking a broader approach to outcome assessment by examining other domains of well-being that extend beyond the domain of PWB. For example, previous research along these lines has reported postintervention improvements in social relationships [52].

### Conclusion

The results of this study provide preliminary support for efforts to invest in refining of existing digital interventions for specialized populations and assessing their efficacy in larger samples, as there is good reason to believe that larger studies will demonstrate efficacy. Robust testing of efficacious digital interventions could allow for rapid, economical, safe, and large-scale dissemination of urgently needed psychological support for frontline staff who are working through the COVID-19 pandemic and its aftermath.

## Appendix

Multimedia Appendix 1 CONSORT-eHEALTH checklist (V 1.6.1).

## Abbreviations

GAD-7: 7-item General Anxiety Disorder
GP: general physician
GQ-6: Gratitude Questionnaire-6
HSCW: health and social care worker
LMM: linear mixed modeling
MH: mental health
MPS: My Possible Self
MT: mental toughness
MTI: Mental Toughness Index
NHS: National Health Service
NHSH: NHS Highland
NHSWBP: NHSH Staff Wellbeing Project
PHQ-9: Patient Health Questionnaire
PPI: patient and public involvement
PWB: psychological well-being
RCT: randomized controlled trial
WEMWBS: Warwick-Edinburgh Mental Well-being Scale
WL: waitlist
