# Supplementary material for: Brief Digital Interventions to Support the Psychological Well-being of NHS Staff During the COVID-19 Pandemic: 3-Arm Pilot Randomized Controlled Trial
Source: JMIR Ment Health. 2022 Apr 4;9(4):e34002. doi: 10.2196/34002 (PMC8982650; doi:10.2196/34002)
Supplement: Multimedia Appendix 1 [file mental_v9i4e34002_app1.pdf]

CONSORT-EHEALTH (V 1.6.1) - Submission/Publication Form

The CONSORT-EHEALTH checklist is intended for authors of randomized trials evaluating web-based and Internet-based applications/interventions, including mobile interventions, electronic games (incl multiplayer games), social media, certain telehealth applications, and other interactive and/or networked electronic applications. Some of the items (e.g. all subitems under item 5 - description of the intervention) may also be applicable for other study designs.

The goal of the CONSORT EHEALTH checklist and guideline is to be
a) a guide for reporting for authors of RCTs,
b) to form a basis for appraisal of an ehealth trial (in terms of validity)

CONSORT-EHEALTH items/subitems are MANDATORY reporting items for studies published in the Journal of Medical Internet Research and other journals / scientific societies endorsing the checklist.

Items numbered 1., 2., 3., 4a., 4b etc are original CONSORT or CONSORT-NPT (non-pharmacologic treatment) items.
Items with Roman numerals (i., ii, iii, iv etc.) are CONSORT-EHEALTH extensions/clarifications.

As the CONSORT-EHEALTH checklist is still considered in a formative stage, we would ask that you also RATE ON A SCALE OF 1-5 how important/useful you feel each item is FOR THE PURPOSE OF THE CHECKLIST and reporting guideline (optional).

Mandatory reporting items are marked with a red *.
In the textboxes, either copy & paste the relevant sections from your manuscript into this form - please include any quotes from your manuscript in QUOTATION MARKS,
or answer directly by providing additional information not in the manuscript, or elaborating on why the item was not relevant for this study.

YOUR ANSWERS WILL BE PUBLISHED AS A SUPPLEMENTARY FILE TO YOUR PUBLICATION IN JMIR AND ARE CONSIDERED PART OF YOUR PUBLICATION (IF ACCEPTED).
Please fill in these questions diligently. Information will not be copyedited, so please use proper spelling and grammar, use correct capitalization, and avoid abbreviations.

DO NOT FORGET TO SAVE AS PDF _AND_ CLICK THE SUBMIT BUTTON SO YOUR ANSWERS ARE IN OUR DATABASE !!!

Citation Suggestion (if you append the pdf as Appendix we suggest to cite this paper in the caption):
Eysenbach G, CONSORT-EHEALTH Group
CONSORT-EHEALTH: Improving and Standardizing Evaluation Reports of Web-based and Mobile Health Interventions
J Med Internet Res 2011;13(4):e126
URL: http://www.jmir.org/2011/4/e126/

doi: 10.2196/jmir.1923
PMID: 22209829

Sign in to Google to save your progress. Learn more

* Required

Your name *

First Last

Johannes H. De Kock

Primary Affiliation (short), City, Country *

University of Toronto, Toronto, Canada

NHS Highland, Inverness, UK

Your e-mail address *

abc@gmail.com

hannes.de@nhs.scot

Title of your manuscript *

Provide the (draft) title of your manuscript.

Brief digital interventions to support the psychological well-being of NHS staff during the COVID-19 pandemic: a three-arm pilot randomised controlled trial

Name of your App/Software/Intervention *

If there is a short and a long/alternate name, write the short name first and add the long name in brackets.

NHSWBP NHS Well-being project

Evaluated Version (if any)

e.g. "V1", "Release 2017-03-01", "Version 2.0.27913"

V.1.6.1

Language(s) *

What language is the intervention/app in? If multiple languages are available, separate by comma (e.g. "English, French")

English

URL of your Intervention Website or App

e.g. a direct link to the mobile app on app in appstore (itunes, Google Play), or URL of the website. If the intervention is a DVD or hardware, you can also link to an Amazon page.

https://www.cso.scot.nhs.uk/wp-content/uploads/COVUHI2001-1.pdf (It was a pilot study - r

URL of an image/screenshot (optional)

Your answer

Accessibility *

Can an enduser access the intervention presently?

- ○ access is free and open
- ○ access only for special usergroups, not open
- ○ access is open to everyone, but requires payment/subscription/in-app purchases
- ◉ app/intervention no longer accessible
- ○ Other:

Primary Medical Indication/Disease/Condition *

e.g. "Stress", "Diabetes", or define the target group in brackets after the condition, e.g. "Autism (Parents of children with)", "Alzheimers (Informal Caregivers of)"

Stress (Health and Social Care staff working tr

Primary Outcomes measured in trial *

comma-separated list of primary outcomes reported in the trial

Depression, Anxiety, Well-being

Secondary/other outcomes

Are there any other outcomes the intervention is expected to affect?

Mental Toughness, Gratitude

Recommended "Dose" *

What do the instructions for users say on how often the app should be used?

- (selected) Approximately Daily
- Approximately Weekly
- Approximately Monthly
- Approximately Yearly
- "as needed"
- Other:

Approx. Percentage of Users (starters) still using the app as recommended after 3 months *

- (selected) unknown / not evaluated
- 0-10%
- 11-20%
- 21-30%
- 31-40%
- 41-50%
- 51-60%
- 61-70%
- 71%-80%
- 81-90%
- 91-100%
- Other:

Overall, was the app/intervention effective? *

- ○ yes: all primary outcomes were significantly better in intervention group vs control
- ○ partly: SOME primary outcomes were significantly better in intervention group vs control
- ○ no statistically significant difference between control and intervention
- ○ potentially harmful: control was significantly better than intervention in one or more outcomes
- ◉ inconclusive: more research is needed
- ○ Other:

Article Preparation Status/Stage *

At which stage in your article preparation are you currently (at the time you fill in this form)

- ○ not submitted yet - in early draft status
- ◉ not submitted yet - in late draft status, just before submission
- ○ submitted to a journal but not reviewed yet
- ○ submitted to a journal and after receiving initial reviewer comments
- ○ submitted to a journal and accepted, but not published yet
- ○ published
- ○ Other:

Journal *

If you already know where you will submit this paper (or if it is already submitted), please provide the journal name (if it is not JMIR, provide the journal name under "other")

- ○ not submitted yet / unclear where I will submit this
- ○ Journal of Medical Internet Research (JMIR)
- ○ JMIR mHealth and UHealth
- ○ JMIR Serious Games
- ◉ JMIR Mental Health
- ○ JMIR Public Health
- ○ JMIR Formative Research
- ○ Other JMIR sister journal
- ○ Other:

Is this a full powered effectiveness trial or a pilot/feasibility trial? *

- ◉ Pilot/feasibility
- ○ Fully powered

Manuscript tracking number *

If this is a JMIR submission, please provide the manuscript tracking number under "other" (The ms tracking number can be found in the submission acknowledgement email, or when you login as author in JMIR. If the paper is already published in JMIR, then the ms tracking number is the four-digit number at the end of the DOI, to be found at the bottom of each published article in JMIR)

- ○ no ms number (yet) / not (yet) submitted to / published in JMIR
- ◉ Other: JMIR Mental Health Manuscript #34002

TITLE AND ABSTRACT

1a) TITLE: Identification as a randomized trial in the title

1a) Does your paper address CONSORT item 1a? *

I.e does the title contain the phrase "Randomized Controlled Trial"? (if not, explain the reason under "other")

- (●) yes
- ( ) Other:

1a-i) Identify the mode of delivery in the title

Identify the mode of delivery. Preferably use “web-based” and/or “mobile” and/or “electronic game” in the title. Avoid ambiguous terms like “online”, “virtual”, “interactive”. Use “Internet-based” only if Intervention includes non-web-based Internet components (e.g. email), use “computer-based” or “electronic” only if offline products are used. Use “virtual” only in the context of “virtual reality” (3-D worlds). Use “online” only in the context of “online support groups”. Complement or substitute product names with broader terms for the class of products (such as “mobile” or “smart phone” instead of “iphone”), especially if the application runs on different platforms.

	1	2	3	4	5	
subitem not at all important	( )	( )	( )	(●)	( )	essential

Clear selection

Does your paper address subitem 1a-i? *

Copy and paste relevant sections from manuscript title (include quotes in quotation marks "like this" to indicate direct quotes from your manuscript), or elaborate on this item by providing additional information not in the ms, or briefly explain why the item is not applicable/relevant for your study

Brief digital interventions: "NHS Highland (NHSH) frontline staff volunteers (N = 169) were randomly assigned to the newly developed NHSH
Staff Wellbeing Project (NHSWBP), an established digital intervention (My Possible Self; MPS), or to a waitlist (WL) condition
for four weeks"

1a-ii) Non-web-based components or important co-interventions in title

Mention non-web-based components or important co-interventions in title, if any (e.g., “with telephone support”).

	1	2	3	4	5	
subitem not at all important	○	○	◉	○	○	essential

Clear selection

Does your paper address subitem 1a-ii?

Copy and paste relevant sections from manuscript title (include quotes in quotation marks "like this" to indicate direct quotes from your manuscript), or elaborate on this item by providing additional information not in the ms, or briefly explain why the item is not applicable/relevant for your study

Yes - we had a wait-list: "NHS Highland (NHSH) frontline staff...or to a waitlist (WL) condition for four weeks"

1a-iii) Primary condition or target group in the title

Mention primary condition or target group in the title, if any (e.g., “for children with Type I Diabetes”) Example: A Web-based and Mobile Intervention with Telephone Support for Children with Type I Diabetes: Randomized Controlled Trial

	1	2	3	4	5	
subitem not at all important	○	○	○	○	◉	essential

Clear selection

Does your paper address subitem 1a-iii? *

Copy and paste relevant sections from manuscript title (include quotes in quotation marks "like this" to indicate direct quotes from your manuscript), or elaborate on this item by providing additional information not in the ms, or briefly explain why the item is not applicable/relevant for your study

Yes: Title: "NHS Staff during the pandemic" Abstract: "NHS Highland (NHSH) frontline staff volunteers"

1b) ABSTRACT: Structured summary of trial design, methods, results, and conclusions

NPT extension: Description of experimental treatment, comparator, care providers, centers, and blinding status.

1b-i) Key features/functionalities/components of the intervention and comparator in the METHODS section of the ABSTRACT

Mention key features/functionalities/components of the intervention and comparator in the abstract. If possible, also mention theories and principles used for designing the site. Keep in mind the needs of systematic reviewers and indexers by including important synonyms. (Note: Only report in the abstract what the main paper is reporting. If this information is missing from the main body of text, consider adding it)

	1	2	3	4	5	
subitem not at all important	○	○	○	◉	○	essential

Clear selection

Does your paper address subitem 1b-i? *

Copy and paste relevant sections from the manuscript abstract (include quotes in quotation marks "like this" to indicate direct quotes from your manuscript), or elaborate on this item by providing additional information not in the ms, or briefly explain why the item is not applicable/relevant for your study

Yes: "Methods: NHS Highland (NHSH) frontline staff volunteers (N = 169) were randomly assigned to the newly developed NHSH
Staff Wellbeing Project (NHSWBP), an established digital intervention (My Possible Self; MPS), or to a waitlist (WL) condition
for four weeks. Attempts were made to blind participants to which digital intervention they were allocated. The interventions
were fully automated, without any human input or guidance. We measured five self-reported psychological outcomes over three
time points: before (baseline), middle (after 2 weeks) and after treatment (4 weeks). The primary outcomes were anxiety
(GAD-7), depression (PHQ-9) and mental well-being (Warwick-Edinburgh Mental Well-being Scale). The secondary outcomes
included mental toughness (Mental Toughness Index) and gratitude (the Gratitude Questionnaire)."

1b-ii) Level of human involvement in the METHODS section of the ABSTRACT

Clarify the level of human involvement in the abstract, e.g., use phrases like “fully automated” vs. “therapist/nurse/care provider/physician-assisted” (mention number and expertise of providers involved, if any). (Note: Only report in the abstract what the main paper is reporting. If this information is missing from the main body of text, consider adding it)

	1	2	3	4	5	
subitem not at all important	○	○	○	◉	○	essential

Clear selection

Does your paper address subitem 1b-ii?

Copy and paste relevant sections from the manuscript abstract (include quotes in quotation marks "like this" to indicate direct quotes from your manuscript), or elaborate on this item by providing additional information not in the ms, or briefly explain why the item is not applicable/relevant for your study

Yes : "The interventions
were fully automated, without any human input or guidance. "

1b-iii) Open vs. closed, web-based (self-assessment) vs. face-to-face assessments in the METHODS section of the ABSTRACT

Mention how participants were recruited (online vs. offline), e.g., from an open access website or from a clinic or a closed online user group (closed usergroup trial), and clarify if this was a purely web-based trial, or there were face-to-face components (as part of the intervention or for assessment). Clearly say if outcomes were self-assessed through questionnaires (as common in web-based trials). Note: In traditional offline trials, an open trial (open-label trial) is a type of clinical trial in which both the researchers and participants know which treatment is being administered. To avoid confusion, use “blinded” or “unblinded” to indicated the level of blinding instead of “open”, as “open” in web-based trials usually refers to “open access” (i.e. participants can self-enrol). (Note: Only report in the abstract what the main paper is reporting. If this information is missing from the main body of text, consider adding it)

	1	2	3	4	5	
subitem not at all important	○	○	◉	○	○	essential

Clear selection

Does your paper address subitem 1b-iii?

Copy and paste relevant sections from the manuscript abstract (include quotes in quotation marks "like this" to indicate direct quotes from your manuscript), or elaborate on this item by providing additional information not in the ms, or briefly explain why the item is not applicable/relevant for your study

yes: "Methods: NHS Highland (NHSH) frontline staff volunteers (N = 169) were randomly assigned to the newly developed NHSH
Staff Wellbeing Project (NHSWBP), an established digital intervention (My Possible Self; MPS), or to a waitlist (WL) condition
for four weeks. Attempts were made to blind participants to which digital intervention they were allocated. The interventions
were fully automated, without any human input or guidance. We measured five self-reported psychological outcomes over three
time points: before (baseline), middle (after 2 weeks) and after treatment (4 weeks). The primary outcomes were anxiety
(GAD-7), depression (PHQ-9) and mental well-being (Warwick-Edinburgh Mental Well-being Scale). The secondary outcomes
included mental toughness (Mental Toughness Index) and gratitude (the Gratitude Questionnaire)"

1b-iv) RESULTS section in abstract must contain use data

Report number of participants enrolled/assessed in each group, the use/uptake of the intervention (e.g., attrition/adherence metrics, use over time, number of logins etc.), in addition to primary/secondary outcomes. (Note: Only report in the abstract what the main paper is reporting. If this information is missing from the main body of text, consider adding it)

	1	2	3	4	5	
subitem not at all important	○	○	○	○	◉	essential

Clear selection

Does your paper address subitem 1b-iv?

Copy and paste relevant sections from the manuscript abstract (include quotes in quotation marks "like this" to indicate direct quotes from your manuscript), or elaborate on this item by providing additional information not in the ms, or briefly explain why the item is not applicable/relevant for your study

Yes: "Results: Retention rates at middle and post-intervention were 77% (n = 130) and 63.3% (n = 107), respectively. At postintervention, small differences were noted between the WL condition and the two treatment conditions on anxiety (vs. MPS: d = .07, 95% CI: -.20, .33; vs. NHSWBP: d = .06, 95% CI: -.19, .31), depression (vs. MPS: d = .37, 95% CI: .07, .66; vs. NHSWBP: d = .18, 95% CI: -.11, .46), and mental well-being (vs. MPS: d = -.04, 95% CI: -.62, -.08; vs. NHSWBP: d = -.15, 95% CI: -.41, .10). A similar pattern of between-group differences was found for the secondary outcomes. The NHSWBP group generally had larger within group effects than the other groups and displayed a greater rate of change compared to the other conditions on all outcomes, except for gratitude, where the rate of change was greatest for the MPS group."

1b-v) CONCLUSIONS/DISCUSSION in abstract for negative trials

Conclusions/Discussions in abstract for negative trials: Discuss the primary outcome - if the trial is negative (primary outcome not changed), and the intervention was not used, discuss whether negative results are attributable to lack of uptake and discuss reasons. (Note: Only report in the abstract what the main paper is reporting. If this information is missing from the main body of text, consider adding it)

	1	2	3	4	5	
subitem not at all important	○	○	○	○	◉	essential

Clear selection

Does your paper address subitem 1b-v?

Copy and paste relevant sections from the manuscript abstract (include quotes in quotation marks "like this" to indicate direct quotes from your manuscript), or elaborate on this item by providing additional information not in the ms, or briefly explain why the item is not applicable/relevant for your study

Yes: "Conclusions: Our analyses provided encouraging results for the use of brief digital psychological interventions in improving psychological well-being among health and social care workers. Future multi-site RCTs, with power to reliably detect differences, are needed to determine the efficacy of contextualised interventions relative to existing digital treatments."

INTRODUCTION

2a) In INTRODUCTION: Scientific background and explanation of rationale

2a-i) Problem and the type of system/solution

Describe the problem and the type of system/solution that is object of the study: intended as stand-alone intervention vs. incorporated in broader health care program? Intended for a particular patient population? Goals of the intervention, e.g., being more cost-effective to other interventions, replace or complement other solutions? (Note: Details about the intervention are provided in "Methods" under 5)

	1	2	3	4	5	
subitem not at all important	○	○	○	●	○	essential

Clear selection

Does your paper address subitem 2a-i? *

Copy and paste relevant sections from the manuscript (include quotes in quotation marks "like this" to indicate direct quotes from your manuscript), or elaborate on this item by providing additional information not in the ms, or briefly explain why the item is not applicable/relevant for your study

Yes: "Mental health (MH) has been deteriorating both globally and across the UK during the coronavirus disease 2019
(COVID-19) pandemic, with large scale population studies reporting increased prevalence of depression and anxiety [1].
There are concerns that the public health crisis has disproportionately impacted the well-being of specialized populations,
including health and social care workers (HSCWs) who provide valuable healthcare services. HSCWs exhibited high
levels of pre-existing mental health problems before the COVID-19 pandemic [2] [3-5], and recent evidence suggests
that this group is at increased risk of experiencing worsening MH outcomes as a direct result of the COVID-19 pandemic
[6, 7] [8, 9]. "Interventions designed to improve MH and psychological well-being (PWB) could help to mitigate the adverse effects of
the COVID-19 pandemic on the well-being of HSCWs [8]. Digital psychological interventions overcome social
distancing, rurality and already overburdened clinician time constraints. Furthermore, digital interventions have a low
cost relative to traditional psychological interventions, they have already been widely used [14], are generally popular
with users and can be accessed anonymously at the user's convenience. Evidence based and rigorously tested digital
interventions could allow for a rapid, economical, and large-scale dissemination of urgently needed psychological
support for frontline staff working through the COVID-19 pandemic and its aftermath."

2a-ii) Scientific background, rationale: What is known about the (type of) system

Scientific background, rationale: What is known about the (type of) system that is the object of the study (be sure to discuss the use of similar systems for other conditions/diagnoses, if appropiate), motivation for the study, i.e. what are the reasons for and what is the context for this specific study, from which stakeholder viewpoint is the study performed, potential impact of findings [2]. Briefly justify the choice of the comparator.

	1	2	3	4	5	
subitem not at all important	○	○	○	◉	○	essential

Clear selection

Does your paper address subitem 2a-ii? *

Copy and paste relevant sections from the manuscript (include quotes in quotation marks "like this" to indicate direct quotes from your manuscript), or elaborate on this item by providing additional information not in the ms, or briefly explain why the item is not applicable/relevant for your study

Yes: "The last decade has seen digital psychological interventions being tested and validated in controlled and long-term follow
up studies, and the number of mobile MH interventions that are available is increasing rapidly. User reports indicate a
significant increase in these apps' downloaded during the first year of the COVID-19 pandemic in the UK [15] and in the
US [14]. Although validated digital interventions have been shown to be clinically efficacious, with effect sizes similar to
that of traditional or face-to-face therapy[16], there is little research into the efficacy of such treatment approaches for
frontline HSCWs who have been working through the COVID-19 pandemic [17]. Furthermore, the majority of digital psychological interventions during the public health crisis have been focused on decreasing symptoms associated with
psychopathology (i.e., depression & anxiety); few have been designed with end-user input (patient and public
involvement; PPI) and oriented toward enhancing PWB[17]. Given the unprecedented scale of the COVID-19
pandemic's burden on HSCWs, specialized and contextual interventions are needed to support the MH of this population.
[18]"

2b) In INTRODUCTION: Specific objectives or hypotheses

Does your paper address CONSORT subitem 2b? *

Copy and paste relevant sections from the manuscript (include quotes in quotation marks "like this" to indicate direct quotes from your manuscript), or elaborate on this item by providing additional information not in the ms, or briefly explain why the item is not applicable/relevant for your study

Yes: "The current study
This study aims to provide preliminary evidence on the use of digital psychological interventions to support frontline
staff psychological health in the context of the COVID-19 pandemic. In a pilot randomised controlled trial (RCT), we
evaluated the use of two smartphone apps designed to support PWB against a control condition (waitlist): 1) My Possible
Self (MPS)[19], which is a well-established validated app with a track record of showing significant improvements in
depression, anxiety and stress in users over a short period of time [19], together with good user satisfaction rates; 2) the
NHS Highland Wellbeing Project (NHSWBP), which is a PPI-informed, brief, fully automated, context (COVID-19
pandemic) and population-specific (frontline staff) digital psychological intervention built on the MPS model and
wireframe to promote psychological well-being among HSCWs.
We predicted that symptoms of depression and anxiety would decline among users randomly allocated to receive digital
psychological interventions, whilst mental well-being would increase, relative to the WL condition. Two positive
psychology concepts shown to mitigate the negative effects of depression and anxiety and promote positive adaptation in
the face of adversity (such as what frontline staff are facing while working through a pandemic), that are amenable to
change, are mental toughness (MT) [20] and gratitude [21]. We also predicted that use of digital psychological
interventions would increase MT and gratitude. Although we predicted both digital interventions to yield improvements
relative to the WL condition, we expected that NHSWBP would show greater rates of improvements because it is
designed specifically for the COVID-19 context. To our knowledge, this is the first trial to examine fully automated, brief
digital psychological interventions aimed to support the psychological health of frontline staff working through the
COVID-19 pandemic."

METHODS

3a) Description of trial design (such as parallel, factorial) including allocation ratio

Does your paper address CONSORT subitem 3a? *

Copy and paste relevant sections from the manuscript (include quotes in quotation marks "like this" to indicate direct quotes from your manuscript), or elaborate on this item by providing additional information not in the ms, or briefly explain why the item is not applicable/relevant for your study

Yes: "parallel-arm pilot randomised controlled trial (RCT)" (see also abstract)

3b) Important changes to methods after trial commencement (such as eligibility criteria), with reasons

Does your paper address CONSORT subitem 3b? *

Copy and paste relevant sections from the manuscript (include quotes in quotation marks "like this" to indicate direct quotes from your manuscript), or elaborate on this item by providing additional information not in the ms, or briefly explain why the item is not applicable/relevant for your study

No changes were needed in this pilot study once the trail commenced

3b-i) Bug fixes, Downtimes, Content Changes

Bug fixes, Downtimes, Content Changes: ehealth systems are often dynamic systems. A description of changes to methods therefore also includes important changes made on the intervention or comparator during the trial (e.g., major bug fixes or changes in the functionality or content) (5-iii) and other “unexpected events” that may have influenced study design such as staff changes, system failures/downtimes, etc. [2].

	1	2	3	4	5	
subitem not at all important	◉	○	○	○	○	essential

Clear selection

Does your paper address subitem 3b-i?

Copy and paste relevant sections from the manuscript (include quotes in quotation marks "like this" to indicate direct quotes from your manuscript), or elaborate on this item by providing additional information not in the ms, or briefly explain why the item is not applicable/relevant for your study

No changes were needed in this pilot study once the trail commenced

4a) Eligibility criteria for participants

Does your paper address CONSORT subitem 4a? *

Copy and paste relevant sections from the manuscript (include quotes in quotation marks "like this" to indicate direct quotes from your manuscript), or elaborate on this item by providing additional information not in the ms, or briefly explain why the item is not applicable/relevant for your study

Yes: "Eligibility criteria
Participants were required to meet the following criteria: UK resident; aged 18 and over; working in NHSH as a health or
social care worker during the COVID-19 pandemic; and own an internet enabled mobile phone. Both “clinical” (doctors,
nurses, allied health professionals) and “non-clinical” (such as administrators) staff were eligible".

4a-i) Computer / Internet literacy

Computer / Internet literacy is often an implicit “de facto” eligibility criterion - this should be explicitly clarified.

	1	2	3	4	5	
subitem not at all important	○	○	○	◉	○	essential

Clear selection

Does your paper address subitem 4a-i?

Copy and paste relevant sections from the manuscript (include quotes in quotation marks "like this" to indicate direct quotes from your manuscript), or elaborate on this item by providing additional information not in the ms, or briefly explain why the item is not applicable/relevant for your study

Yes: "Participants were required to meet the following criteria: UK resident; aged 18 and over; working in NHSH as a health or
social care worker during the COVID-19 pandemic; and own an internet enabled mobile phone."

4a-ii) Open vs. closed, web-based vs. face-to-face assessments:

Open vs. closed, web-based vs. face-to-face assessments: Mention how participants were recruited (online vs. offline), e.g., from an open access website or from a clinic, and clarify if this was a purely web-based trial, or there were face-to-face components (as part of the intervention or for assessment), i.e., to what degree got the study team to know the participant. In online-only trials, clarify if participants were quasi-anonymous and whether having multiple identities was possible or whether technical or logistical measures (e.g., cookies, email confirmation, phone calls) were used to detect/prevent these.

	1	2	3	4	5	
subitem not at all important	○	○	○	◉	○	essential

Clear selection

Does your paper address subitem 4a-ii? *

Copy and paste relevant sections from the manuscript (include quotes in quotation marks "like this" to indicate direct quotes from your manuscript), or elaborate on this item by providing additional information not in the ms, or briefly explain why the item is not applicable/relevant for your study

Yes: "Participants were recruited
locally and online between July 2020 and September 2020. Data collection took place at the beginning, middle and end
of the pilot RCT intervention phase, which ran from September 2020 to October 2020. Recruitment was
conducted digitally by NHSH Human Resources, which included emails and electronic newsletters. Further recruitment
was conducted via GP practice managers, as well as heads of departments in primary and secondary care. A secondary level of recruitment was conducted on social media; a page for the study was made on Twitter, Facebook and LinkedIn.
Paid advertisements were also used on Facebook and LinkedIn to promote the study. Across all recruitment routes,
interested individuals were directed to a secure data collection website via a weblink where they first reviewed
information about the study and gave electronic consent to participate.

"Randomisation
A research assistant not involved in the RCT randomised participants after baseline using computerised
Simple randomization. Allocation was either to the MPS, NHSWBP, or WL condition. Participants received advice of
their group assignment by email. Participants were blinded to which intervention they received by styling the two
interventions, and communications to participants similarly. Participants downloaded the same app from the
iTunes/Appstore/Playstore and a code was sent back to them to initiate the intervention that they received. "

4a-iii) Information giving during recruitment

Information given during recruitment. Specify how participants were briefed for recruitment and in the informed consent procedures (e.g., publish the informed consent documentation as appendix, see also item X26), as this information may have an effect on user self-selection, user expectation and may also bias results.

	1	2	3	4	5	
subitem not at all important	○	○	○	◉	○	essential

Clear selection

Does your paper address subitem 4a-iii?

Copy and paste relevant sections from the manuscript (include quotes in quotation marks "like this" to indicate direct quotes from your manuscript), or elaborate on this item by providing additional information not in the ms, or briefly explain why the item is not applicable/relevant for your study

Yes: "Randomisation
A research assistant not involved in the RCT randomised participants after baseline using computerised
Simple randomization. Allocation was either to the MPS, NHSWBP, or WL condition. Participants received advice of
their group assignment by email. Participants were blinded to which intervention they received by styling the two
interventions, and communications to participants similarly. Participants downloaded the same app from the
iTunes/Appstore/Playstore and a code was sent back to them to initiate the intervention that they received."

"Recruitment was
conducted digitally by NHSH Human Resources, which included emails and electronic newsletters. Further recruitment
was conducted via GP practice managers, as well as heads of departments in primary and secondary care. A secondary level of recruitment was conducted on social media; a page for the study was made on Twitter, Facebook and LinkedIn.
Paid advertisements were also used on Facebook and LinkedIn to promote the study. Across all recruitment routes,
interested individuals were directed to a secure data collection website via a weblink where they first reviewed
information about the study and gave electronic consent to participate. Eligible participants then completed a baseline
survey, after which they were randomized to a condition. All participants were asked to complete follow-up surveys after
the first two weeks of the intervention (middle) and four weeks after baseline following completion of the intervention
period. At each assessment point, participants accessed the survey via a weblink sent to them in an email message"

4b) Settings and locations where the data were collected

Does your paper address CONSORT subitem 4b? *

Copy and paste relevant sections from the manuscript (include quotes in quotation marks "like this" to indicate direct quotes from your manuscript), or elaborate on this item by providing additional information not in the ms, or briefly explain why the item is not applicable/relevant for your study

Yes "Across all recruitment routes,
interested individuals were directed to a secure data collection website via a weblink where they first reviewed
information about the study and gave electronic consent to participate. Eligible participants then completed a baseline
survey, after which they were randomized to a condition. All participants were asked to complete follow-up surveys after
the first two weeks of the intervention (middle) and four weeks after baseline following completion of the intervention
period. At each assessment point, participants accessed the survey via a weblink sent to them in an email message.

" This study was a part of the Scottish Government’s Rapid Research into COVID-19 and time restrictions limited
recruitment activities; it was not possible to extend recruitment activities or product development beyond the grant’s
funding timeframe. Written informed consent was provided by all participants. The RCT was approved by the NHS
Health Research Authority (20/SW/0098) and registered at ISRCTN18107122. The intervention phase ran from
September 7 to October 5 2020, during the start of the second wave of the COVID-19 pandemic in Scotland."

4b-i) Report if outcomes were (self-)assessed through online questionnaires

Clearly report if outcomes were (self-)assessed through online questionnaires (as common in web-based trials) or otherwise.

	1	2	3	4	5	
subitem not at all important	○	○	●	○	○	essential

Clear selection

Does your paper address subitem 4b-i? *

Copy and paste relevant sections from the manuscript (include quotes in quotation marks "like this" to indicate direct quotes from your manuscript), or elaborate on this item by providing additional information not in the ms, or briefly explain why the item is not applicable/relevant for your study

Yes: "All participants were asked to complete follow-up surveys after
the first two weeks of the intervention (middle) and four weeks after baseline following completion of the intervention
period. At each assessment point, participants accessed the survey via a weblink sent to them in an email message."

4b-ii) Report how institutional affiliations are displayed

Report how institutional affiliations are displayed to potential participants [on ehealth media], as affiliations with prestigious hospitals or universities may affect volunteer rates, use, and reactions with regards to an intervention.(Not a required item – describe only if this may bias results)

	1	2	3	4	5	
subitem not at all important	◉	○	○	○	○	essential

Clear selection

Does your paper address subitem 4b-ii?

Copy and paste relevant sections from the manuscript (include quotes in quotation marks "like this" to indicate direct quotes from your manuscript), or elaborate on this item by providing additional information not in the ms, or briefly explain why the item is not applicable/relevant for your study

This was not deemed important for our study

5) The interventions for each group with sufficient details to allow replication, including how and when they were actually administered

5-i) Mention names, credential, affiliations of the developers, sponsors, and owners

Mention names, credential, affiliations of the developers, sponsors, and owners [6] (if authors/evaluators are owners or developer of the software, this needs to be declared in a “Conflict of interest” section or mentioned elsewhere in the manuscript).

	1	2	3	4	5	
subitem not at all important	(●)	○	○	○	○	essential

Clear selection

Does your paper address subitem 5-i?

Copy and paste relevant sections from the manuscript (include quotes in quotation marks "like this" to indicate direct quotes from your manuscript), or elaborate on this item by providing additional information not in the ms, or briefly explain why the item is not applicable/relevant for your study

not applicable to our study

5-ii) Describe the history/development process

Describe the history/development process of the application and previous formative evaluations (e.g., focus groups, usability testing), as these will have an impact on adoption/use rates and help with interpreting results.

	1	2	3	4	5	
subitem not at all important	○	○	○	○	(●)	essential

Clear selection

Does your paper address subitem 5-ii?

Copy and paste relevant sections from the manuscript (include quotes in quotation marks "like this" to indicate direct quotes from your manuscript), or elaborate on this item by providing additional information not in the ms, or briefly explain why the item is not applicable/relevant for your study

Yes: "NHS Highland Staff Wellbeing Project (NHSWBP)
The NHSWBP is a PPI-informed, brief, fully automated, context- (COVID-19) and population-specific (NHSH frontline
staff) digital psychological intervention (smartphone app) based on MPS. It utilises the tried and tested cognitive
behavioural therapeutic (CBT) and positive psychological techniques delivered via the MPS [19] smartphone app's
modules. There were a number of ways in which the NHSWBP app differed from the MPS app. First, the NHSWBP was presented as a coherent narrative with a fictional character, a Scottish nurse named Iona, who guided participants through
the linear narrative of the app and its interventions. Participants also received automated text messages from Iona to
engage them in the overall narrative and to motivate continued engagement with the intervention. Second, the NHSWBP
was designed following PPI feedback, which included input about which MPS modules were most relevant, the duration
of the modules, and the coherence and flow of the presentation format. Third, the NHSWBP provided links to local and
national 24-hour support services. Similarly to MPS, participants were able to monitor and record their mood and levels
of distress or well-being, add notes, and identify and record triggers for low mood and anxiety. The intervention lasted
for four weeks and consisted of two parts: Part 1 (duration two weeks) focused on increasing participants' happiness,
resilience and well-being; and Part 2 (duration two weeks) focused on managing low mood and anxiety effectively. The
NHSWBP was co-designed by the University of the Highlands and Islands (UHI), NHSH and the software and technical
team that supports the MPS app. The NHSWBP was designed using the MPS app platform and participant
communication system, owing to its established track record and NHS approval. "

5-iii) Revisions and updating

Revisions and updating. Clearly mention the date and/or version number of the application/intervention (and comparator, if applicable) evaluated, or describe whether the intervention underwent major changes during the evaluation process, or whether the development and/or content was "frozen" during the trial. Describe dynamic components such as news feeds or changing content which may have an impact on the replicability of the intervention (for unexpected events see item 3b).

	1	2	3	4	5	
subitem not at all important	◉	○	○	○	○	essential

Clear selection

Does your paper address subitem 5-iii?

Copy and paste relevant sections from the manuscript (include quotes in quotation marks "like this" to indicate direct quotes from your manuscript), or elaborate on this item by providing additional information not in the ms, or briefly explain why the item is not applicable/relevant for your study

No - this was a pilot study for a pilot project that only had 1 version.

5-iv) Quality assurance methods

Provide information on quality assurance methods to ensure accuracy and quality of information provided [1], if applicable.

	1	2	3	4	5	
subitem not at all important	○	○	◉	○	○	essential

Clear selection

Does your paper address subitem 5-iv?

Copy and paste relevant sections from the manuscript (include quotes in quotation marks "like this" to indicate direct quotes from your manuscript), or elaborate on this item by providing additional information not in the ms, or briefly explain why the item is not applicable/relevant for your study

Yes, in the primary and secondary outcomes section we provide a rationale for using assessments that are seen as the most widely standards for each construct.

5-v) Ensure replicability by publishing the source code, and/or providing screenshots/screen-capture video, and/or providing flowcharts of the algorithms used

Ensure replicability by publishing the source code, and/or providing screenshots/screen-capture video, and/or providing flowcharts of the algorithms used. Replicability (i.e., other researchers should in principle be able to replicate the study) is a hallmark of scientific reporting.

	1	2	3	4	5	
subitem not at all important	(●)	( )	( )	( )	( )	essential

Clear selection

Does your paper address subitem 5-v?

Copy and paste relevant sections from the manuscript (include quotes in quotation marks "like this" to indicate direct quotes from your manuscript), or elaborate on this item by providing additional information not in the ms, or briefly explain why the item is not applicable/relevant for your study

This was a pilot study and we aim to due provide source code, screen shots etc in the full-scale RCT

5-vi) Digital preservation

Digital preservation: Provide the URL of the application, but as the intervention is likely to change or disappear over the course of the years; also make sure the intervention is archived (Internet Archive, webcitation.org, and/or publishing the source code or screenshots/videos alongside the article). As pages behind login screens cannot be archived, consider creating demo pages which are accessible without login.

	1	2	3	4	5	
subitem not at all important	(●)	( )	( )	( )	( )	essential

Clear selection

Does your paper address subitem 5-vi?

Copy and paste relevant sections from the manuscript (include quotes in quotation marks "like this" to indicate direct quotes from your manuscript), or elaborate on this item by providing additional information not in the ms, or briefly explain why the item is not applicable/relevant for your study

This was a pilot study intervention is no longer available for download and as closed only to participants.

5-vii) Access

Access: Describe how participants accessed the application, in what setting/context, if they had to pay (or were paid) or not, whether they had to be a member of specific group. If known, describe how participants obtained “access to the platform and Internet” [1]. To ensure access for editors/reviewers/readers, consider to provide a “backdoor” login account or demo mode for reviewers/readers to explore the application (also important for archiving purposes, see vi).

	1	2	3	4	5	
subitem not at all important	○	○	○	◉	○	essential

Clear selection

Does your paper address subitem 5-vii? *

Copy and paste relevant sections from the manuscript (include quotes in quotation marks "like this" to indicate direct quotes from your manuscript), or elaborate on this item by providing additional information not in the ms, or briefly explain why the item is not applicable/relevant for your study

Yes: "Eligible participants then completed a baseline
survey, after which they were randomized to a condition. All participants were asked to complete follow-up surveys after
the first two weeks of the intervention (middle) and four weeks after baseline following completion of the intervention
period. At each assessment point, participants accessed the survey via a weblink sent to them in an email message."

"Allocation was either to the MPS, NHSWBP, or WL condition. Participants received advice of their group assignment by email. Participants were blinded to which intervention they received by styling the two
interventions, and communications to participants similarly. Participants downloaded the same app from the
iTunes/Appstore/Playstore and a code was sent back to them to initiate the intervention that they received".

5-viii) Mode of delivery, features/functionalities/components of the intervention and comparator, and the theoretical framework

Describe mode of delivery, features/functionalities/components of the intervention and comparator, and the theoretical framework [6] used to design them (instructional strategy [1], behaviour change techniques, persuasive features, etc., see e.g., [7, 8] for terminology). This includes an in-depth description of the content (including where it is coming from and who developed it) [1]," whether [and how] it is tailored to individual circumstances and allows users to track their progress and receive feedback" [6]. This also includes a description of communication delivery channels and – if computer-mediated communication is a component – whether communication was synchronous or asynchronous [6]. It also includes information on presentation strategies [1], including page design principles, average amount of text on pages, presence of hyperlinks to other resources, etc. [1].

	1	2	3	4	5	
subitem not at all important	○	○	○	◉	○	essential

Clear selection

Does your paper address subitem 5-viii? *

Copy and paste relevant sections from the manuscript (include quotes in quotation marks "like this" to indicate direct quotes from your manuscript), or elaborate on this item by providing additional information not in the ms, or briefly explain why the item is not applicable/relevant for your study

Yes: "Interventions
My Possible Self (MPS)
MPS is a tried and tested, NHS approved [22] smartphone well-being app with a validated track record of showing
significant improvements in depression, anxiety and stress in its users over a short period of time [19]. It is fully
automated and freely available to NHS staff. This intervention has modules that cover a variety of topics and can be
accessed in any order, including coping effectively with depression and anxiety, enhancing happiness, improving sleep
quality and practicing mindfulness.
NHS Highland Staff Wellbeing Project (NHSWBP)
The NHSWBP is a PPI-informed, brief, fully automated, context- (COVID-19) and population-specific (NHSH frontline
staff) digital psychological intervention (smartphone app) based on MPS. It utilises the tried and tested cognitive
behavioural therapeutic (CBT) and positive psychological techniques delivered via the MPS [19] smartphone app's
modules. There were a number of ways in which the NHSWBP app differed from the MPS app. First, the NHSWBP was presented as a coherent narrative with a fictional character, a Scottish nurse named Iona, who guided participants through
the linear narrative of the app and its interventions. Participants also received automated text messages from Iona to
engage them in the overall narrative and to motivate continued engagement with the intervention. Second, the NHSWBP
was designed following PPI feedback, which included input about which MPS modules were most relevant, the duration
of the modules, and the coherence and flow of the presentation format. Third, the NHSWBP provided links to local and
national 24-hour support services. Similarly to MPS, participants were able to monitor and record their mood and levels
of distress or well-being, add notes, and identify and record triggers for low mood and anxiety. The intervention lasted
for four weeks and consisted of two parts: Part 1 (duration two weeks) focused on increasing participants' happiness,
resilience and well-being; and Part 2 (duration two weeks) focused on managing low mood and anxiety effectively. The
NHSWBP was co-designed by the University of the Highlands and Islands (UHI), NHSH and the software and technical
team that supports the MPS app. The NHSWBP was designed using the MPS app platform and participant
communication system, owing to its established track record and NHS approval.

5-ix) Describe use parameters

Describe use parameters (e.g., intended “doses” and optimal timing for use). Clarify what instructions or recommendations were given to the user, e.g., regarding timing, frequency, heaviness of use, if any, or was the intervention used ad libitum.

	1	2	3	4	5	
subitem not at all important	○	○	◉	○	○	essential

Clear selection

Does your paper address subitem 5-ix?

Copy and paste relevant sections from the manuscript (include quotes in quotation marks "like this" to indicate direct quotes from your manuscript), or elaborate on this item by providing additional information not in the ms, or briefly explain why the item is not applicable/relevant for your study

Yes: NHS Highland Staff Wellbeing Project (NHSWBP)
The NHSWBP is a PPI-informed, brief, fully automated, context- (COVID-19) and population-specific (NHSH frontline
staff) digital psychological intervention (smartphone app) based on MPS. It utilises the tried and tested cognitive
behavioural therapeutic (CBT) and positive psychological techniques delivered via the MPS [19] smartphone app's
modules. There were a number of ways in which the NHSWBP app differed from the MPS app. First, the NHSWBP was
presented as a coherent narrative with a fictional character, a Scottish nurse named Iona, who guided participants through
the linear narrative of the app and its interventions. Participants also received automated text messages from Iona to
engage them in the overall narrative and to motivate continued engagement with the intervention. Second, the NHSWBP
was designed following PPI feedback, which included input about which MPS modules were most relevant, the duration
of the modules, and the coherence and flow of the presentation format. Third, the NHSWBP provided links to local and
national 24-hour support services. Similarly to MPS, participants were able to monitor and record their mood and levels
of distress or well-being, add notes, and identify and record triggers for low mood and anxiety. The intervention lasted
for four weeks and consisted of two parts: Part 1 (duration two weeks) focused on increasing participants' happiness,
resilience and well-being; and Part 2 (duration two weeks) focused on managing low mood and anxiety effectively. The
NHSWBP was co-designed by the University of the Highlands and Islands (UHI), NHSH and the software and technical
team that supports the MPS app. The NHSWBP was designed using the MPS app platform and participant
communication system, owing to its established track record and NHS approval.

5-x) Clarify the level of human involvement

Clarify the level of human involvement (care providers or health professionals, also technical assistance) in the e-intervention or as co-intervention (detail number and expertise of professionals involved, if any, as well as “type of assistance offered, the timing and frequency of the support, how it is initiated, and the medium by which the assistance is delivered”. It may be necessary to distinguish between the level of human involvement required for the trial, and the level of human involvement required for a routine application outside of a RCT setting (discuss under item 21 – generalizability).

	1	2	3	4	5	
subitem not at all important	○	○	○	◉	○	essential

Clear selection

Does your paper address subitem 5-x?

Copy and paste relevant sections from the manuscript (include quotes in quotation marks "like this" to indicate direct quotes from your manuscript), or elaborate on this item by providing additional information not in the ms, or briefly explain why the item is not applicable/relevant for your study

Yes: "The interventions
were fully automated, without any human input or guidance."

5-xi) Report any prompts/reminders used

Report any prompts/reminders used: Clarify if there were prompts (letters, emails, phone calls, SMS) to use the application, what triggered them, frequency etc. It may be necessary to distinguish between the level of prompts/reminders required for the trial, and the level of prompts/reminders for a routine application outside of a RCT setting (discuss under item 21 – generalizability).

	1	2	3	4	5	
subitem not at all important	○	○	○	◉	○	essential

Clear selection

Does your paper address subitem 5-xi? *

Copy and paste relevant sections from the manuscript (include quotes in quotation marks "like this" to indicate direct quotes from your manuscript), or elaborate on this item by providing additional information not in the ms, or briefly explain why the item is not applicable/relevant for your study

Yes: "Yes: NHS Highland Staff Wellbeing Project (NHSWBP)
The NHSWBP is a PPI-informed, brief, fully automated, context- (COVID-19) and population-specific (NHSH frontline
staff) digital psychological intervention (smartphone app) based on MPS. It utilises the tried and tested cognitive
behavioural therapeutic (CBT) and positive psychological techniques delivered via the MPS [19] smartphone app's
modules. There were a number of ways in which the NHSWBP app differed from the MPS app. First, the NHSWBP was
presented as a coherent narrative with a fictional character, a Scottish nurse named Iona, who guided participants through
the linear narrative of the app and its interventions. Participants also received automated text messages from Iona to
engage them in the overall narrative and to motivate continued engagement with the intervention. Second, the NHSWBP
was designed following PPI feedback, which included input about which MPS modules were most relevant, the duration
of the modules, and the coherence and flow of the presentation format. Third, the NHSWBP provided links to local and
national 24-hour support services. Similarly to MPS, participants were able to monitor and record their mood and levels
of distress or well-being, add notes, and identify and record triggers for low mood and anxiety. The intervention lasted
for four weeks and consisted of two parts: Part 1 (duration two weeks) focused on increasing participants' happiness,
resilience and well-being; and Part 2 (duration two weeks) focused on managing low mood and anxiety effectively. The
NHSWBP was co-designed by the University of the Highlands and Islands (UHI), NHSH and the software and technical
team that supports the MPS app. The NHSWBP was designed using the MPS app platform and participant
communication system, owing to its established track record and NHS approval. "

5-xii) Describe any co-interventions (incl. training/support)

Describe any co-interventions (incl. training/support): Clearly state any interventions that are provided in addition to the targeted eHealth intervention, as ehealth intervention may not be designed as stand-alone intervention. This includes training sessions and support [1]. It may be necessary to distinguish between the level of training required for the trial, and the level of training for a routine application outside of a RCT setting (discuss under item 21 – generalizability.

	1	2	3	4	5	
subitem not at all important	(•)	○	○	○	○	essential

Clear selection

Does your paper address subitem 5-xii? *

Copy and paste relevant sections from the manuscript (include quotes in quotation marks "like this" to indicate direct quotes from your manuscript), or elaborate on this item by providing additional information not in the ms, or briefly explain why the item is not applicable/relevant for your study

No ancillary training/support were used in this pilot study

6a) Completely defined pre-specified primary and secondary outcome measures, including how and when they were assessed

Does your paper address CONSORT subitem 6a? *

Copy and paste relevant sections from the manuscript (include quotes in quotation marks "like this" to indicate direct quotes from your manuscript), or elaborate on this item by providing additional information not in the ms, or briefly explain why the item is not applicable/relevant for your study

Yes: " All participants were asked to complete follow-up surveys after
the first two weeks of the intervention (middle) and four weeks after baseline following completion of the intervention
period. At each assessment point, participants accessed the survey via a weblink sent to them in an email message." Furthermore a full description of the Primary and secondary outcomes are provided, including their online use during the COVID-19 pandemic.

6a-i) Online questionnaires: describe if they were validated for online use and apply CHERRIES items to describe how the questionnaires were designed/deployed

If outcomes were obtained through online questionnaires, describe if they were validated for online use and apply CHERRIES items to describe how the questionnaires were designed/deployed [9].

	1	2	3	4	5	
subitem not at all important	○	○	○	◉	○	essential

Clear selection

Does your paper address subitem 6a-i?

Copy and paste relevant sections from manuscript text

Yes: A full a full description of the Primary and secondary outcomes are provided, including their online use during the COVID-19 pandemic.
"Primary outcomes
Post-intervention was the primary timepoint for all outcomes.
Depression. The Patient Health Questionnaire (PHQ-9)[23] was used to measure depression. The 9 items ask participants
to consider how bothered they have been over the past two weeks according to each statement (e.g., “feeling tired or
having little energy”). The questionnaire score ranges from 0-27; each question is given a four-point response (“Not at
all” = 0, “Nearly every day” = 3). The questionnaire has demonstrated diagnostic validity [23]. This measure has been
used extensively in the UK [24] and internationally [25] to measure levels of depression in various population settings
during the COVID-19 pandemic.
Anxiety. The 7-item General Anxiety Disorder (GAD-7)[26] scale was used to measure anxiety. Similar to the PHQ-9,
each item asks the respondent to consider the statement based on how much they have been bothered over a two-week
period (e.g., “Feeling nervous anxious or on edge”). Each item is scaled from 0 (“Not at all”) to 3 (“Nearly every day”)
with a total score range of 0-21. A number of studies during the COVID-19 pandemic have used the GAD-7 to measure
levels of anxiety in various UK and international population settings, including in frontline staff working through this
pandemic [8, 24].
Mental Well-being. Mental well-being was measured using the Warwick-Edinburgh Mental Well-being Scale
(WEMWBS) [27]. The scale consists of 14-items used to measure subjective well-being and psychological functioning.
The wording of each item is positive and aimed to address positive aspects of MH. Responses are completed using a 5-
point scale (1 = “None of the time”, 5 = “All of the time”); the total score ranges from 14-70. WEMWBS has been
validated for use in the UK [27], and has been used internationally [28] and in the UK [29] to measure the MWB of
HSCWs during this pandemic."

6a-ii) Describe whether and how “use” (including intensity of use/dosage) was defined/measured/monitored

Describe whether and how “use” (including intensity of use/dosage) was defined/measured/monitored (logins, logfile analysis, etc.). Use/adoption metrics are important process outcomes that should be reported in any ehealth trial.

	1	2	3	4	5	
subitem not at all important	○	○	◉	○	○	essential

Clear selection

Does your paper address subitem 6a-ii?

Copy and paste relevant sections from manuscript text

Yes: "Program adherence
Adherence, defined as the extent to which participants engaged with the intervention, was examined for both the
NHSWBP and MPS groups with respect to average interactions per user. Adherence was deemed to be good for
both digital interventions with participants in the NHSWBP group interacted on average 37.4 times with the intervention (more than once per day on average) during the month long intervention, whilst the MPS group
interacted on average 37.5 times. None of the adherence indices correlated with demographic, clinical history
and primary and secondary outcome data obtained at baseline. No harmful or unintended effects were reported
by the participants."

6a-iii) Describe whether, how, and when qualitative feedback from participants was obtained

Describe whether, how, and when qualitative feedback from participants was obtained (e.g., through emails, feedback forms, interviews, focus groups).

	1	2	3	4	5	
subitem not at all important	◉	○	○	○	○	essential

Clear selection

Does your paper address subitem 6a-iii?

Copy and paste relevant sections from manuscript text

Qualitative feedback was not a part of this study. We did however design this with public involvement and had specific test- groups providing us with qualitative feedback outside of this study.

6b) Any changes to trial outcomes after the trial commenced, with reasons

Does your paper address CONSORT subitem 6b? *

Copy and paste relevant sections from the manuscript (include quotes in quotation marks "like this" to indicate direct quotes from your manuscript), or elaborate on this item by providing additional information not in the ms, or briefly explain why the item is not applicable/relevant for your study

No changes were required in this pilot project

7a) How sample size was determined

NPT: When applicable, details of whether and how the clustering by care provides or centers was addressed

7a-i) Describe whether and how expected attrition was taken into account when calculating the sample size

Describe whether and how expected attrition was taken into account when calculating the sample size.

	1	2	3	4	5	
subitem not at all important	(●)	○	○	○	○	essential

Clear selection

Does your paper address subitem 7a-i?

Copy and paste relevant sections from manuscript title (include quotes in quotation marks "like this" to indicate direct quotes from your manuscript), or elaborate on this item by providing additional information not in the ms, or briefly explain why the item is not applicable/relevant for your study

Yes:

"Given that this was a pilot trial being done in a limited time, the sample size targets were based on pragmatic factors
rather than an expectation of having the power to enable detection of the expected effect sizes"
"Post-hoc power calculation
Instead of using the observed effect size to calculate post-hoc study power (which could introduce bias)[45], we
used the observed sample size and a fixed threshold for power and significance, and calculated the smallest
effect size that could be reliably detected with our sample size. By using this approach, together with our study
design, we found that our study could detect an effect size of at least f = 0.27 at 80% power"

7b) When applicable, explanation of any interim analyses and stopping guidelines

Does your paper address CONSORT subitem 7b? *

Copy and paste relevant sections from the manuscript (include quotes in quotation marks "like this" to indicate direct quotes from your manuscript), or elaborate on this item by providing additional information not in the ms, or briefly explain why the item is not applicable/relevant for your study

Yes: We explain that this is a limited pilot study that lasted 4 weeks only

8a) Method used to generate the random allocation sequence

NPT: When applicable, how care providers were allocated to each trial group

Does your paper address CONSORT subitem 8a? *

Copy and paste relevant sections from the manuscript (include quotes in quotation marks "like this" to indicate direct quotes from your manuscript), or elaborate on this item by providing additional information not in the ms, or briefly explain why the item is not applicable/relevant for your study

Yes: "Randomisation
A research assistant not involved in the RCT randomised participants after baseline using computerised
Simple randomization. "

8b) Type of randomisation; details of any restriction (such as blocking and block size)

Does your paper address CONSORT subitem 8b? *

Copy and paste relevant sections from the manuscript (include quotes in quotation marks "like this" to indicate direct quotes from your manuscript), or elaborate on this item by providing additional information not in the ms, or briefly explain why the item is not applicable/relevant for your study

Yes: "Randomisation
A research assistant not involved in the RCT randomised participants after baseline using computerised
Simple randomization. "

9) Mechanism used to implement the random allocation sequence (such as sequentially numbered containers), describing any steps taken to conceal the sequence until interventions were assigned

Does your paper address CONSORT subitem 9? *

Copy and paste relevant sections from the manuscript (include quotes in quotation marks "like this" to indicate direct quotes from your manuscript), or elaborate on this item by providing additional information not in the ms, or briefly explain why the item is not applicable/relevant for your study

Yes: "Randomisation
A research assistant not involved in the RCT randomised participants after baseline using computerised
Simple randomization. Allocation was either to the MPS, NHSWBP, or WL condition. Participants received advice of
their group assignment by email. Participants were blinded to which intervention they received by styling the two
interventions, and communications to participants similarly. Participants downloaded the same app from the
iTunes/Appstore/Playstore and a code was sent back to them to initiate the intervention that they received. "

10) Who generated the random allocation sequence, who enrolled participants, and who assigned participants to interventions

Does your paper address CONSORT subitem 10? *

Copy and paste relevant sections from the manuscript (include quotes in quotation marks "like this" to indicate direct quotes from your manuscript), or elaborate on this item by providing additional information not in the ms, or briefly explain why the item is not applicable/relevant for your study

Yes: Yes: "Randomisation
A research assistant not involved in the RCT randomised participants after baseline using computerised
Simple randomization. Allocation was either to the MPS, NHSWBP, or WL condition. Participants received advice of
their group assignment by email. Participants were blinded to which intervention they received by styling the two
interventions, and communications to participants similarly. Participants downloaded the same app from the
iTunes/Appstore/Playstore and a code was sent back to them to initiate the intervention that they received. "

11a) If done, who was blinded after assignment to interventions (for example, participants, care providers, those assessing outcomes) and how

NPT: Whether or not administering co-interventions were blinded to group assignment

11a-i) Specify who was blinded, and who wasn't

Specify who was blinded, and who wasn't. Usually, in web-based trials it is not possible to blind the participants [1, 3] (this should be clearly acknowledged), but it may be possible to blind outcome assessors, those doing data analysis or those administering co-interventions (if any).

	1	2	3	4	5	
subitem not at all important	○	○	◉	○	○	essential

Clear selection

Does your paper address subitem 11a-i? *

Copy and paste relevant sections from the manuscript (include quotes in quotation marks "like this" to indicate direct quotes from your manuscript), or elaborate on this item by providing additional information not in the ms, or briefly explain why the item is not applicable/relevant for your study

Yes: Yes: "Randomisation
A research assistant not involved in the RCT randomised participants after baseline using computerised
Simple randomization. Allocation was either to the MPS, NHSWBP, or WL condition. Participants received advice of
their group assignment by email. Participants were blinded to which intervention they received by styling the two
interventions, and communications to participants similarly. Participants downloaded the same app from the
iTunes/Appstore/Playstore and a code was sent back to them to initiate the intervention that they received. "

11a-ii) Discuss e.g., whether participants knew which intervention was the "intervention of interest" and which one was the "comparator"

Informed consent procedures (4a-ii) can create biases and certain expectations - discuss e.g., whether participants knew which intervention was the "intervention of interest" and which one was the "comparator".

	1	2	3	4	5	
subitem not at all important	○	○	○	◉	○	essential

Clear selection

Does your paper address subitem 11a-ii?

Copy and paste relevant sections from the manuscript (include quotes in quotation marks "like this" to indicate direct quotes from your manuscript), or elaborate on this item by providing additional information not in the ms, or briefly explain why the item is not applicable/relevant for your study

Yes: Yes: "Randomisation
A research assistant not involved in the RCT randomised participants after baseline using computerised
Simple randomization. Allocation was either to the MPS, NHSWBP, or WL condition. Participants received advice of
their group assignment by email. Participants were blinded to which intervention they received by styling the two
interventions, and communications to participants similarly. Participants downloaded the same app from the
iTunes/Appstore/Playstore and a code was sent back to them to initiate the intervention that they received. "

11b) If relevant, description of the similarity of interventions

(this item is usually not relevant for ehealth trials as it refers to similarity of a placebo or sham intervention to a active medication/intervention)

Does your paper address CONSORT subitem 11b? *

Copy and paste relevant sections from the manuscript (include quotes in quotation marks "like this" to indicate direct quotes from your manuscript), or elaborate on this item by providing additional information not in the ms, or briefly explain why the item is not applicable/relevant for your study

Yes: Under the intervention heading we describe this in detail:
"Interventions
My Possible Self (MPS)
MPS is a tried and tested, NHS approved [22] smartphone well-being app with a validated track record of showing
significant improvements in depression, anxiety and stress in its users over a short period of time [19]. It is fully
automated and freely available to NHS staff. This intervention has modules that cover a variety of topics and can be
accessed in any order, including coping effectively with depression and anxiety, enhancing happiness, improving sleep
quality and practicing mindfulness.
NHS Highland Staff Wellbeing Project (NHSWBP)
The NHSWBP is a PPI-informed, brief, fully automated, context- (COVID-19) and population-specific (NHSH frontline
staff) digital psychological intervention (smartphone app) based on MPS. It utilises the tried and tested cognitive
behavioural therapeutic (CBT) and positive psychological techniques delivered via the MPS [19] smartphone app's
modules. There were a number of ways in which the NHSWBP app differed from the MPS app. First, the NHSWBP was presented as a coherent narrative with a fictional character, a Scottish nurse named Iona, who guided participants through
the linear narrative of the app and its interventions. Participants also received automated text messages from Iona to
engage them in the overall narrative and to motivate continued engagement with the intervention. Second, the NHSWBP
was designed following PPI feedback, which included input about which MPS modules were most relevant, the duration
of the modules, and the coherence and flow of the presentation format. Third, the NHSWBP provided links to local and
national 24-hour support services. Similarly to MPS, participants were able to monitor and record their mood and levels
of distress or well-being, add notes, and identify and record triggers for low mood and anxiety. The intervention lasted
for four weeks and consisted of two parts: Part 1 (duration two weeks) focused on increasing participants' happiness,
resilience and well-being; and Part 2 (duration two weeks) focused on managing low mood and anxiety effectively. The
NHSWBP was co-designed by the University of the Highlands and Islands (UHI), NHSH and the software and technical
team that supports the MPS app. The NHSWBP was designed using the MPS app platform and participant
communication system, owing to its established track record and NHS approval."

12a) Statistical methods used to compare groups for primary and secondary outcomes

NPT: When applicable, details of whether and how the clustering by care providers or centers was addressed

Does your paper address CONSORT subitem 12a? *

Copy and paste relevant sections from the manuscript (include quotes in quotation marks "like this" to indicate direct quotes from your manuscript), or elaborate on this item by providing additional information not in the ms, or briefly explain why the item is not applicable/relevant for your study

Yes: "Statistical analyses
Statistical analyses and data manipulations were implemented with R[37]. Baseline characteristics of participants
randomly allocated to the three intervention groups were compared using chi square. The effects of the MPS and
NHSWBP interventions on psychological measures were examined using intention-to-treat (ITT) analyses that included
data from all participants who completed the baseline assessment and any follow-up assessment. No imputation was used
for missing data. Standard regression models assume independent observations. To adequately account for the
dependencies in the data, we adopted the linear mixed modelling (LMM) approach [38] for the analyses of the data. This
approach is appropriate for studying the relationships and sources of variation in the dataset. It uses all available data and
efficiently handles missing data, thereby avoiding listwise deletion. LMM models all sources of variation in the data and
avoids the need for data imputation. Each psychological outcome was modelled as a function of time, treatment group,
and their interaction and adjusting for random effects due to individual differences and repeated observations from each
participant. The models allow for each participant to have a different trajectory. Model parameters were estimated using
restricted maximum likelihood. The best model was selected using likelihood ratio test. Based on the chosen model,
marginal means were estimated and multiple comparison of group by time interaction tests conducted using sets of Tukey
adjusted interaction contrasts [39]; degrees of freedom was calculated using Kenward-Roger [40] .
The effects were tested at a significance level of 0.05, adjusted depending on the number of contrasts in multiple tests.
Cohen's d was calculated by standardising the mean difference of within and between groups using the square root of the
sum of all the variance components from the mixed models. This is to adequately represent the study design and account
for all sources of variation in data [41, 42].
Linear regression slopes of each psychological measure were modelled as a function of time, treatment and timetreatment interaction. Pairs of the slopes were then compared using the lsmean approach of Lenth (2016) to determine the
intervention that brought about higher rate of change in the mean of the psychological measures [43]. This analysis used
data for the three-time period and models the average trend for each of the measured outcomes. A second analysis
adjusted for the baseline by entering the baseline values of the outcome of interest as a covariate in the mixed effect
model that also included group by time intervention as fixed effect."

12a-i) Imputation techniques to deal with attrition / missing values

Imputation techniques to deal with attrition / missing values: Not all participants will use the intervention/comparator as intended and attrition is typically high in ehealth trials. Specify how participants who did not use the application or dropped out from the trial were treated in the statistical analysis (a complete case analysis is strongly discouraged, and simple imputation techniques such as LOCF may also be problematic [4]).

	1	2	3	4	5	
subitem not at all important	○	○	○	○	◉	essential

Clear selection

Does your paper address subitem 12a-i? *

Copy and paste relevant sections from the manuscript (include quotes in quotation marks "like this" to indicate direct quotes from your manuscript), or elaborate on this item by providing additional information not in the ms, or briefly explain why the item is not applicable/relevant for your study

Yes: "Statistical analyses
Statistical analyses and data manipulations were implemented with R[37]. Baseline characteristics of participants
randomly allocated to the three intervention groups were compared using chi square. The effects of the MPS and
NHSWBP interventions on psychological measures were examined using intention-to-treat (ITT) analyses that included
data from all participants who completed the baseline assessment and any follow-up assessment. No imputation was used
for missing data. Standard regression models assume independent observations. To adequately account for the
dependencies in the data, we adopted the linear mixed modelling (LMM) approach [38] for the analyses of the data. This
approach is appropriate for studying the relationships and sources of variation in the dataset. It uses all available data and
efficiently handles missing data, thereby avoiding listwise deletion. LMM models all sources of variation in the data and
avoids the need for data imputation. Each psychological outcome was modelled as a function of time, treatment group,
and their interaction and adjusting for random effects due to individual differences and repeated observations from each
participant. The models allow for each participant to have a different trajectory. Model parameters were estimated using
restricted maximum likelihood. The best model was selected using likelihood ratio test. Based on the chosen model,
marginal means were estimated and multiple comparison of group by time interaction tests conducted using sets of Tukey
adjusted interaction contrasts [39]; degrees of freedom was calculated using Kenward-Roger [40] .
The effects were tested at a significance level of 0.05, adjusted depending on the number of contrasts in multiple tests.
Cohen's d was calculated by standardising the mean difference of within and between groups using the square root of the
sum of all the variance components from the mixed models. This is to adequately represent the study design and account
for all sources of variation in data [41, 42].
Linear regression slopes of each psychological measure were modelled as a function of time, treatment and timetreatment interaction. Pairs of the slopes were then compared using the lsmean approach of Lenth (2016) to determine the
intervention that brought about higher rate of change in the mean of the psychological measures [43]. This analysis used
data for the three-time period and models the average trend for each of the measured outcomes. A second analysis
adjusted for the baseline by entering the baseline values of the outcome of interest as a covariate in the mixed effect
model that also included group by time intervention as fixed effect."

12b) Methods for additional analyses, such as subgroup analyses and adjusted analyses

Does your paper address CONSORT subitem 12b? *

Copy and paste relevant sections from the manuscript (include quotes in quotation marks "like this" to indicate direct quotes from your manuscript), or elaborate on this item by providing additional information not in the ms, or briefly explain why the item is not applicable/relevant for your study

Yes: "Statistical analyses
Statistical analyses and data manipulations were implemented with R[37]. Baseline characteristics of participants
randomly allocated to the three intervention groups were compared using chi square. The effects of the MPS and
NHSWBP interventions on psychological measures were examined using intention-to-treat (ITT) analyses that included
data from all participants who completed the baseline assessment and any follow-up assessment. No imputation was used
for missing data. Standard regression models assume independent observations. To adequately account for the
dependencies in the data, we adopted the linear mixed modelling (LMM) approach [38] for the analyses of the data. This
approach is appropriate for studying the relationships and sources of variation in the dataset. It uses all available data and
efficiently handles missing data, thereby avoiding listwise deletion. LMM models all sources of variation in the data and
avoids the need for data imputation. Each psychological outcome was modelled as a function of time, treatment group,
and their interaction and adjusting for random effects due to individual differences and repeated observations from each
participant. The models allow for each participant to have a different trajectory. Model parameters were estimated using
restricted maximum likelihood. The best model was selected using likelihood ratio test. Based on the chosen model,
marginal means were estimated and multiple comparison of group by time interaction tests conducted using sets of Tukey
adjusted interaction contrasts [39]; degrees of freedom was calculated using Kenward-Roger [40] .
The effects were tested at a significance level of 0.05, adjusted depending on the number of contrasts in multiple tests.
Cohen's d was calculated by standardising the mean difference of within and between groups using the square root of the
sum of all the variance components from the mixed models. This is to adequately represent the study design and account
for all sources of variation in data [41, 42].
Linear regression slopes of each psychological measure were modelled as a function of time, treatment and timetreatment interaction. Pairs of the slopes were then compared using the lsmean approach of Lenth (2016) to determine the
intervention that brought about higher rate of change in the mean of the psychological measures [43]. This analysis used
data for the three-time period and models the average trend for each of the measured outcomes. A second analysis
adjusted for the baseline by entering the baseline values of the outcome of interest as a covariate in the mixed effect
model that also included group by time intervention as fixed effect."

X26) REB/IRB Approval and Ethical Considerations [recommended as subheading under "Methods"] (not a CONSORT item)

X26-i) Comment on ethics committee approval

	1	2	3	4	5	
subitem not at all important	○	○	○	○	◉	essential

Clear selection

Does your paper address subitem X26-i?

Copy and paste relevant sections from the manuscript (include quotes in quotation marks "like this" to indicate direct quotes from your manuscript), or elaborate on this item by providing additional information not in the ms, or briefly explain why the item is not applicable/relevant for your study

Yes: "The RCT was approved by the NHS
Health Research Authority (20/SW/0098) and registered at ISRCTN18107122. "

x26-ii) Outline informed consent procedures

Outline informed consent procedures e.g., if consent was obtained offline or online (how? Checkbox, etc.?), and what information was provided (see 4a-ii). See [6] for some items to be included in informed consent documents.

	1	2	3	4	5	
subitem not at all important	○	○	○	◉	○	essential

Clear selection

Does your paper address subitem X26-ii?

Copy and paste relevant sections from the manuscript (include quotes in quotation marks "like this" to indicate direct quotes from your manuscript), or elaborate on this item by providing additional information not in the ms, or briefly explain why the item is not applicable/relevant for your study

Yes: "Across all recruitment routes,
interested individuals were directed to a secure data collection website via a weblink where they first reviewed
information about the study and gave electronic consent to participate."

X26-iii) Safety and security procedures

Safety and security procedures, incl. privacy considerations, and any steps taken to reduce the likelihood or detection of harm (e.g., education and training, availability of a hotline)

	1	2	3	4	5	
subitem not at all important	(●)	( )	( )	( )	( )	essential

Clear selection

Does your paper address subitem X26-iii?

Copy and paste relevant sections from the manuscript (include quotes in quotation marks "like this" to indicate direct quotes from your manuscript), or elaborate on this item by providing additional information not in the ms, or briefly explain why the item is not applicable/relevant for your study

Yes: "the NHSWBP provided links to local and
national 24-hour support services."

RESULTS

13a) For each group, the numbers of participants who were randomly assigned, received intended treatment, and were analysed for the primary outcome

NPT: The number of care providers or centers performing the intervention in each group and the number of patients treated by each care provider in each center

Does your paper address CONSORT subitem 13a? *

Copy and paste relevant sections from the manuscript (include quotes in quotation marks "like this" to indicate direct quotes from your manuscript), or elaborate on this item by providing additional information not in the ms, or briefly explain why the item is not applicable/relevant for your study

Yes: "Details of enrolment into the trial, organised according to the CONSORT guidelines [44], are shown in Figure 1. Of the
225 people who expressed an interest in the study, completed eligibility screening information and provided consent to
participate, 54 (24%) did not complete the pre-intervention questionnaire and 2 declined to participate (1%). These 56
individuals were excluded from the analyses, leaving a study sample of N = 169 participants. The distribution of
participant characteristics at baseline and post-intervention are reported in Table 1."

13b) For each group, losses and exclusions after randomisation, together with reasons

Does your paper address CONSORT subitem 13b? (NOTE: Preferably, this is shown in a CONSORT flow diagram) *

Copy and paste relevant sections from the manuscript (include quotes in quotation marks "like this" to indicate direct quotes from your manuscript), or elaborate on this item by providing additional information not in the ms, or briefly explain why the item is not applicable/relevant for your study

Yes: "Details of enrolment into the trial, organised according to the CONSORT guidelines [44], are shown in Figure 1. Of the
225 people who expressed an interest in the study, completed eligibility screening information and provided consent to
participate, 54 (24%) did not complete the pre-intervention questionnaire and 2 declined to participate (1%). These 56
individuals were excluded from the analyses, leaving a study sample of N = 169 participants. The distribution of
participant characteristics at baseline and post-intervention are reported in Table 1."

13b-i) Attrition diagram

Strongly recommended: An attrition diagram (e.g., proportion of participants still logging in or using the intervention/comparator in each group plotted over time, similar to a survival curve) or other figures or tables demonstrating usage/dose/engagement.

	1	2	3	4	5	
subitem not at all important	○	○	○	◉	○	essential

Clear selection

Does your paper address subitem 13b-i?

Copy and paste relevant sections from the manuscript or cite the figure number if applicable (include quotes in quotation marks "like this" to indicate direct quotes from your manuscript), or elaborate on this item by providing additional information not in the ms, or briefly explain why the item is not applicable/relevant for your study

Yes: "Details of enrolment into the trial, organised according to the CONSORT guidelines [44], are shown in Figure 1. Of the
225 people who expressed an interest in the study, completed eligibility screening information and provided consent to
participate, 54 (24%) did not complete the pre-intervention questionnaire and 2 declined to participate (1%). These 56
individuals were excluded from the analyses, leaving a study sample of N = 169 participants. The distribution of
participant characteristics at baseline and post-intervention are reported in Table 1."

14a) Dates defining the periods of recruitment and follow-up

Does your paper address CONSORT subitem 14a? *

Copy and paste relevant sections from the manuscript (include quotes in quotation marks "like this" to indicate direct quotes from your manuscript), or elaborate on this item by providing additional information not in the ms, or briefly explain why the item is not applicable/relevant for your study

Yes: "Participants were recruited
locally and online between July 2020 and September 2020. Data collection took place at the beginning, middle and end
of the pilot RCT intervention phase, which ran from September 2020 to October 2020. "

"This study was a part of the Scottish Government's Rapid Research into COVID-19 and time restrictions limited
recruitment activities; it was not possible to extend recruitment activities or product development beyond the grant's
funding timeframe. Written informed consent was provided by all participants. The RCT was approved by the NHS
Health Research Authority (20/SW/0098) and registered at ISRCTN18107122. The intervention phase ran from
September 7 to October 5 2020, during the start of the second wave of the COVID-19 pandemic in Scotland.

14a-i) Indicate if critical "secular events" fell into the study period

Indicate if critical "secular events" fell into the study period, e.g., significant changes in Internet resources available or "changes in computer hardware or Internet delivery resources"

	1	2	3	4	5	
subitem not at all important	○	○	○	○	●	essential

Clear selection

Does your paper address subitem 14a-i?

Copy and paste relevant sections from the manuscript (include quotes in quotation marks "like this" to indicate direct quotes from your manuscript), or elaborate on this item by providing additional information not in the ms, or briefly explain why the item is not applicable/relevant for your study

Yes: "Participants were recruited
locally and online between July 2020 and September 2020. Data collection took place at the beginning, middle and end
of the pilot RCT intervention phase, which ran from September 2020 to October 2020. "

"This study was a part of the Scottish Government's Rapid Research into COVID-19 and time restrictions limited
recruitment activities; it was not possible to extend recruitment activities or product development beyond the grant's
funding timeframe. Written informed consent was provided by all participants. The RCT was approved by the NHS
Health Research Authority (20/SW/0098) and registered at ISRCTN18107122. The intervention phase ran from
September 7 to October 5 2020, during the start of the second wave of the COVID-19 pandemic in Scotland.

14b) Why the trial ended or was stopped (early)

Does your paper address CONSORT subitem 14b? *

Copy and paste relevant sections from the manuscript (include quotes in quotation marks "like this" to indicate direct quotes from your manuscript), or elaborate on this item by providing additional information not in the ms, or briefly explain why the item is not applicable/relevant for your study

This was not applicable to our study

15) A table showing baseline demographic and clinical characteristics for each group

NPT: When applicable, a description of care providers (case volume, qualification, expertise, etc.) and centers (volume) in each group

Does your paper address CONSORT subitem 15? *

Copy and paste relevant sections from the manuscript (include quotes in quotation marks "like this" to indicate direct quotes from your manuscript), or elaborate on this item by providing additional information not in the ms, or briefly explain why the item is not applicable/relevant for your study

Yes: "Rates of attrition across the demographic, professional, and clinical characteristics of the
participants are presented in Table 1"

15-i) Report demographics associated with digital divide issues

In ehealth trials it is particularly important to report demographics associated with digital divide issues, such as age, education, gender, social-economic status, computer/Internet/ehealth literacy of the participants, if known.

	1	2	3	4	5	
subitem not at all important	○	○	◉	○	○	essential

Clear selection

Does your paper address subitem 15-i? *

Copy and paste relevant sections from the manuscript (include quotes in quotation marks "like this" to indicate direct quotes from your manuscript), or elaborate on this item by providing additional information not in the ms, or briefly explain why the item is not applicable/relevant for your study

Yes: "Rates of attrition across the demographic, professional, and clinical characteristics of the
participants are presented in Table 1,"

16) For each group, number of participants (denominator) included in each analysis and whether the analysis was by original assigned groups

16-i) Report multiple “denominators” and provide definitions

Report multiple “denominators” and provide definitions: Report N’s (and effect sizes) “across a range of study participation [and use] thresholds” [1], e.g., N exposed, N consented, N used more than x times, N used more than y weeks, N participants “used” the intervention/comparator at specific pre-defined time points of interest (in absolute and relative numbers per group). Always clearly define “use” of the intervention.

	1	2	3	4	5	
subitem not at all important	○	○	○	○	◉	essential

Clear selection

Does your paper address subitem 16-i? *

Copy and paste relevant sections from the manuscript (include quotes in quotation marks "like this" to indicate direct quotes from your manuscript), or elaborate on this item by providing additional information not in the ms, or briefly explain why the item is not applicable/relevant for your study

Yes: Statistical analyses
"Statistical analyses and data manipulations were implemented with R[37]. Baseline characteristics of participants
randomly allocated to the three intervention groups were compared using chi square. The effects of the MPS and
NHSWBP interventions on psychological measures were examined using intention-to-treat (ITT) analyses that included
data from all participants who completed the baseline assessment and any follow-up assessment. No imputation was used
for missing data. Standard regression models assume independent observations. To adequately account for the
dependencies in the data, we adopted the linear mixed modelling (LMM) approach [38] for the analyses of the data. This
approach is appropriate for studying the relationships and sources of variation in the dataset. It uses all available data and
efficiently handles missing data, thereby avoiding listwise deletion. LMM models all sources of variation in the data and
avoids the need for data imputation. Each psychological outcome was modelled as a function of time, treatment group,
and their interaction and adjusting for random effects due to individual differences and repeated observations from each
participant. The models allow for each participant to have a different trajectory. Model parameters were estimated using
restricted maximum likelihood. The best model was selected using likelihood ratio test. Based on the chosen model,
marginal means were estimated and multiple comparison of group by time interaction tests conducted using sets of Tukey
adjusted interaction contrasts [39]; degrees of freedom was calculated using Kenward-Roger [40] .
The effects were tested at a significance level of 0.05, adjusted depending on the number of contrasts in multiple tests.
Cohen's d was calculated by standardising the mean difference of within and between groups using the square root of the
sum of all the variance components from the mixed models. This is to adequately represent the study design and account
for all sources of variation in data [41, 42].
Linear regression slopes of each psychological measure were modelled as a function of time, treatment and timetreatment interaction. Pairs of the slopes were then compared using the lsmean approach of Lenth (2016) to determine the
intervention that brought about higher rate of change in the mean of the psychological measures [43]. This analysis used
data for the three-time period and models the average trend for each of the measured outcomes. A second analysis
adjusted for the baseline by entering the baseline values of the outcome of interest as a covariate in the mixed effect
model that also included group by time intervention as fixed effect."

16-ii) Primary analysis should be intent-to-treat

Primary analysis should be intent-to-treat, secondary analyses could include comparing only “users”, with the appropriate caveats that this is no longer a randomized sample (see 18-i).

	1	2	3	4	5	
subitem not at all important	○	○	○	◉	○	essential

Clear selection

Does your paper address subitem 16-ii?

Copy and paste relevant sections from the manuscript (include quotes in quotation marks "like this" to indicate direct quotes from your manuscript), or elaborate on this item by providing additional information not in the ms, or briefly explain why the item is not applicable/relevant for your study

Yes: "Primary outcomes
Post-intervention was the primary timepoint for all outcomes.
Depression. The Patient Health Questionnaire (PHQ-9)[23] was used to measure depression. The 9 items ask participants
to consider how bothered they have been over the past two weeks according to each statement (e.g., "feeling tired or
having little energy"). The questionnaire score ranges from 0-27; each question is given a four-point response ("Not at
all" = 0, "Nearly every day" = 3). The questionnaire has demonstrated diagnostic validity [23]. This measure has been
used extensively in the UK [24] and internationally [25] to measure levels of depression in various population settings
during the COVID-19 pandemic.
Anxiety. The 7-item General Anxiety Disorder (GAD-7)[26] scale was used to measure anxiety. Similar to the PHQ-9,
each item asks the respondent to consider the statement based on how much they have been bothered over a two-week
period (e.g., "Feeling nervous anxious or on edge"). Each item is scaled from 0 ("Not at all") to 3 ("Nearly every day")
with a total score range of 0-21. A number of studies during the COVID-19 pandemic have used the GAD-7 to measure
levels of anxiety in various UK and international population settings, including in frontline staff working through this
pandemic [8, 24].
Mental Well-being. Mental well-being was measured using the Warwick-Edinburgh Mental Well-being Scale
(WEMWBS) [27]. The scale consists of 14-items used to measure subjective well-being and psychological functioning.
The wording of each item is positive and aimed to address positive aspects of MH. Responses are completed using a 5-
point scale (1 = "None of the time", 5 = "All of the time"); the total score ranges from 14-70. WEMWBS has been
validated for use in the UK [27], and has been used internationally [28] and in the UK [29] to measure the MWB of
HSCWs during this pandemic."

17a) For each primary and secondary outcome, results for each group, and the estimated effect size and its precision (such as 95% confidence interval)

Does your paper address CONSORT subitem 17a? *

Copy and paste relevant sections from the manuscript (include quotes in quotation marks "like this" to indicate direct quotes from your manuscript), or elaborate on this item by providing additional information not in the ms, or briefly explain why the item is not applicable/relevant for your study

Yes: "Outcomes
Table 2 reports the observed mean scores for each outcome at baseline, mid-intervention and post-intervention in the
three treatment groups (see Table S2 for estimated marginal mean scores for individual times and groups). Figure 2
depicts these scores for the three conditions on the primary and secondary outcome measures at baseline, midintervention and post-intervention. "

"Standardised effect size
The between and within-group effect sizes (standardised mean difference) on the primary and secondary
outcomes calculated using observed means are presented in Table 3. At post-intervention, between-group effect
sizes were small to medium for the primary (NHSWBP vs. MPS; d range = .19 to -.20; WL vs. MPS; d range =
-.04 to .36; WL vs. NHSWBP d range =.06 to -.18) and secondary outcome measures. Adjusted contrasts
comparing between-group changes from baseline to post-intervention on all outcomes are reported in Table S3.
The results showed a consistent pattern of greater improvements in depression, anxiety, well-being, MT, and
gratitude among participants in the digital intervention groups (MPS and NHSWBP) at post-intervention
compared to the WL group.
At post-intervention, a small difference was noted between the WL condition and the two treatment
conditions on anxiety (vs. MPS: d = 0.07, 95% CI: -0.20, 0.33; vs. NHSWBP: d = 0.06, 95% CI: -0.19, 0.31),
depression (vs. MPS: d = 0.37, 95% CI: 0.07, 0.66; vs. NHSWBP: d = 0.18, 95% CI: -0.11, 0.46), and mental
well-being (vs. MPS: d =-0.04, 95% CI: -0.62, -0.08; vs. NHSWBP: d = -0.15, 95% CI: -0.41, 0.10). The
NHSWBP group generally had larger within group effects than the other groups. Within-group effects for both
MPS and the NHSWBP groups ranged from small to medium based on observed means (MPS: d range = -.31 to
0.25, NHSWBP: d-range = -.38 to .24). For WL group, within-group effects were generally small for the
primary outcomes (d range = -.12 to .16) and small to medium for the secondary outcome measures (d range
= .13 to .27)."

17a-i) Presentation of process outcomes such as metrics of use and intensity of use

In addition to primary/secondary (clinical) outcomes, the presentation of process outcomes such as metrics of use and intensity of use (dose, exposure) and their operational definitions is critical. This does not only refer to metrics of attrition (13-b) (often a binary variable), but also to more continuous exposure metrics such as “average session length”. These must be accompanied by a technical description how a metric like a “session” is defined (e.g., timeout after idle time) [1] (report under item 6a).

	1	2	3	4	5	
subitem not at all important	○	○	◉	○	○	essential

Clear selection

Does your paper address subitem 17a-i?

Copy and paste relevant sections from the manuscript (include quotes in quotation marks "like this" to indicate direct quotes from your manuscript), or elaborate on this item by providing additional information not in the ms, or briefly explain why the item is not applicable/relevant for your study

Yes: "Program adherence
Adherence, defined as the extent to which participants engaged with the intervention, was examined for both the
NHSWBP and MPS groups with respect to average interactions per user. Adherence was deemed to be good for
both digital interventions with participants in the NHSWBP group interacted on average 37.4 times with theintervention (more than once per day on average) during the month long intervention, whilst the MPS group
interacted on average 37.5 times. None of the adherence indices correlated with demographic, clinical history
and primary and secondary outcome data obtained at baseline. No harmful or unintended effects were reported
by the participants."

17b) For binary outcomes, presentation of both absolute and relative effect sizes is recommended

Does your paper address CONSORT subitem 17b? *

Copy and paste relevant sections from the manuscript (include quotes in quotation marks "like this" to indicate direct quotes from your manuscript), or elaborate on this item by providing additional information not in the ms, or briefly explain why the item is not applicable/relevant for your study

Not applicable to our paper

18) Results of any other analyses performed, including subgroup analyses and adjusted analyses, distinguishing pre-specified from exploratory

Does your paper address CONSORT subitem 18? *

Copy and paste relevant sections from the manuscript (include quotes in quotation marks "like this" to indicate direct quotes from your manuscript), or elaborate on this item by providing additional information not in the ms, or briefly explain why the item is not applicable/relevant for your study

Not applicable to our paper

18-i) Subgroup analysis of comparing only users

A subgroup analysis of comparing only users is not uncommon in ehealth trials, but if done, it must be stressed that this is a self-selected sample and no longer an unbiased sample from a randomized trial (see 16-iii).

	1	2	3	4	5	
subitem not at all important	◉	○	○	○	○	essential

Clear selection

Does your paper address subitem 18-i?

Copy and paste relevant sections from the manuscript (include quotes in quotation marks "like this" to indicate direct quotes from your manuscript), or elaborate on this item by providing additional information not in the ms, or briefly explain why the item is not applicable/relevant for your study

Not applicable to our paper

19) All important harms or unintended effects in each group

(for specific guidance see CONSORT for harms)

Does your paper address CONSORT subitem 19? *

Copy and paste relevant sections from the manuscript (include quotes in quotation marks "like this" to indicate direct quotes from your manuscript), or elaborate on this item by providing additional information not in the ms, or briefly explain why the item is not applicable/relevant for your study

Yes: "No harmful or unintended effects were reported
by the participants."

19-i) Include privacy breaches, technical problems

Include privacy breaches, technical problems. This does not only include physical "harm" to participants, but also incidents such as perceived or real privacy breaches [1], technical problems, and other unexpected/unintended incidents. "Unintended effects" also includes unintended positive effects [2].

	1	2	3	4	5	
subitem not at all important	○	○	◉	○	○	essential

Clear selection

Does your paper address subitem 19-i?

Copy and paste relevant sections from the manuscript (include quotes in quotation marks "like this" to indicate direct quotes from your manuscript), or elaborate on this item by providing additional information not in the ms, or briefly explain why the item is not applicable/relevant for your study

Yes: Yes: "No harmful or unintended effects were reported
by the participants."

19-ii) Include qualitative feedback from participants or observations from staff/researchers

Include qualitative feedback from participants or observations from staff/researchers, if available, on strengths and shortcomings of the application, especially if they point to unintended/unexpected effects or uses. This includes (if available) reasons for why people did or did not use the application as intended by the developers.

	1	2	3	4	5	
subitem not at all important	○	○	◉	○	○	essential

Clear selection

Does your paper address subitem 19-ii?

Copy and paste relevant sections from the manuscript (include quotes in quotation marks "like this" to indicate direct quotes from your manuscript), or elaborate on this item by providing additional information not in the ms, or briefly explain why the item is not applicable/relevant for your study

Yes: Even though the qualitative aspects of our study was a separate part of the study. We had specific groups testing and providing feedback feedback, also for the design of a larger fully powered RCT.

"We conducted a novel pilot RCT to evaluate two brief, fully automated digital health interventions in a
sample of frontline staff working through the second wave of the COVID-19 pandemic. The trial proceeded
successfully during challenging circumstances in the shadow of the second wave of the COVID-19 pandemic in
the UK. Our low cost study demonstrated that it was possible to recruit 169 people working in a small NHS
Board within a short duration and deliver a technically innovative intervention on a modest financial budget.
The NHSWBP app was designed with end-user (PPI) input and worked well throughout, with good adherence
and no major flaws or bugs, nor evidence of harm reported by the participants. Furthermore, the WL control
design was effective at retaining participants (who otherwise might have lost interest in the study and dropped
out if it was just a no-treatment control rather than WL). We have also accumulated rich background data that
could assist in identifying the possible drivers of drop-out, which could be used to modify the design of the
intervention to improve retention in a larger future trial. Although this was a pilot trial that we conducted during
a prescribed limited time with a relatively small sample size, the findings of this study provide encouraging
results for future full trials of digital psychological interventions that are designed to support the MH and PWB
of HSCWs who are working under conditions of extreme stress."

DISCUSSION

22) Interpretation consistent with results, balancing benefits and harms, and considering other relevant evidence

NPT: In addition, take into account the choice of the comparator, lack of or partial blinding, and unequal expertise of care providers or centers in each group

22-i) Restate study questions and summarize the answers suggested by the data, starting with primary outcomes and process outcomes (use)

Restate study questions and summarize the answers suggested by the data, starting with primary outcomes and process outcomes (use).

	1	2	3	4	5	
subitem not at all important	○	○	○	○	◉	essential

Clear selection

Does your paper address subitem 22-i? *

Copy and paste relevant sections from the manuscript (include quotes in quotation marks "like this" to indicate direct quotes from your manuscript), or elaborate on this item by providing additional information not in the ms, or briefly explain why the item is not applicable/relevant for your study

Yes: "There are three key findings of interest in this study. First, the primary outcomes investigated showed
decreases in levels depression (NHSWBP & MPS) and anxiety (NHSWBP) when compared to the WL group.
The rate of decrease in depression and anxiety symptoms was the greatest among those exposed to the
NHSWBP intervention. Our results also indicate that the individuals exposed to the digital interventions and WL
conditions experienced an increase in mental well-being with the rate of increase again shown to be the greatest
for those exposed to the NHSWBP intervention. "

22-ii) Highlight unanswered new questions, suggest future research

Highlight unanswered new questions, suggest future research.

	1	2	3	4	5	
subitem not at all important	○	○	○	○	◉	essential

Clear selection

Does your paper address subitem 22-ii?

Copy and paste relevant sections from the manuscript (include quotes in quotation marks "like this" to indicate direct quotes from your manuscript), or elaborate on this item by providing additional information not in the ms, or briefly explain why the item is not applicable/relevant for your study

Yes: "Thirdly, our results also provide preliminary support for the development or modification of digital
interventions to be context specific as the NHSWBP showed the greater rates of symptom improvement of the
two interventions tested. Future trials assessing context specific digital interventions for specialized populations
in larger samples are warranted, as there is good reason to believe those larger studies will demonstrate efficacy.
The digital nature of these interventions was seen to be safe, cost effective and rapidly modifiable to context.
The future application of similar, context specific robustly tested interventions could be scalable to other
contexts with mental health human resources needs [49]."

20) Trial limitations, addressing sources of potential bias, imprecision, and, if relevant, multiplicity of analyses

20-i) Typical limitations in ehealth trials

Typical limitations in ehealth trials: Participants in ehealth trials are rarely blinded. Ehealth trials often look at a multiplicity of outcomes, increasing risk for a Type I error. Discuss biases due to non-use of the intervention/usability issues, biases through informed consent procedures, unexpected events.

	1	2	3	4	5	
subitem not at all important	○	○	○	○	●	essential

Clear selection

Does your paper address subitem 20-i? *

Copy and paste relevant sections from the manuscript (include quotes in quotation marks "like this" to indicate direct quotes from your manuscript), or elaborate on this item by providing additional information not in the ms, or briefly explain why the item is not applicable/relevant for your study

Yes: "Limitations and future research directions
There are several limitations of this study that need to be acknowledged. First, participants included a small
sample of HSCWs from a single NHS site. Although the vast majority of respondents were female, this does not
differ dramatically from the gender composition of the whole HSCW workforce in NHS Highland [50]. As our
objective was to gather preliminary evidence on the potential benefits of two digital interventions in this
population, the study was not powered as an efficacy trial and so confidence intervals around estimated effects
were wide (indicating the small sample may have contributed to statistical uncertainty) and the findings may not
be generalizable to other populations and HSCWs living in other contexts. Second, the treatment period was
restricted to four weeks, and it is possible that changes in MH and PWB require more engagement in the
intervention materials. In addition, some outcomes may change more gradually and require a longer period to
improve. For example, gratitude exercises can orientate a person to experience more grateful emotions, but it
could take more than four weeks for changes in dispositional gratitude to emerge. Future research would do well
to track and monitor whether gains that are made during treatment are maintained or change over time. Third,
the MPS app was publicly available for download throughout the duration of our study, and participants were
not restricted to use other modalities or medications during this pilot which raises the possibility that treatment
effects might be cross-contaminated. Fourth, the attrition rate at post-intervention was 36.7%. The dropout rate
was lowest in WL group, which is likely attributable to participants waiting to receive either of the digital
interventions. Although we did not find any substantial evidence of attrition bias, it is possible that participants
who dropped out from the intervention groups were less satisfied with the program or experienced less than
positive outcomes. Additional research is needed to explore the mechanisms underlying the effects that emerged
in this study and to identify the relative contributions of the components that comprised each of the digital
interventions. There may also be value in taking a broader approach to outcome assessment by examining other
domains of well-being that extend beyond the domain of PWB. For example, previous research along these lines
has reported post-intervention improvements in social relationships [51]. "

21) Generalisability (external validity, applicability) of the trial findings

NPT: External validity of the trial findings according to the intervention, comparators, patients, and care providers or centers involved in the trial

21-i) Generalizability to other populations

Generalizability to other populations: In particular, discuss generalizability to a general Internet population, outside of a RCT setting, and general patient population, including applicability of the study results for other organizations

	1	2	3	4	5	
subitem not at all important	○	○	○	○	●	essential

Clear selection

Does your paper address subitem 21-i?

Copy and paste relevant sections from the manuscript (include quotes in quotation marks "like this" to indicate direct quotes from your manuscript), or elaborate on this item by providing additional information not in the ms, or briefly explain why the item is not applicable/relevant for your study

Yes: "As our
objective was to gather preliminary evidence on the potential benefits of two digital interventions in this
population, the study was not powered as an efficacy trial and so confidence intervals around estimated effects
were wide (indicating the small sample may have contributed to statistical uncertainty) and the findings may not
be generalizable to other populations and HSCWs living in other contexts"

"Future trials assessing context specific digital interventions for specialized populations in larger samples are warranted, as there is good reason to believe those larger studies will demonstrate efficacy.
The digital nature of these interventions was seen to be safe, cost effective and rapidly modifiable to context.
The future application of similar, context specific robustly tested interventions could be scalable to other
contexts with mental health human resources needs "

21-ii) Discuss if there were elements in the RCT that would be different in a routine application setting

Discuss if there were elements in the RCT that would be different in a routine application setting (e.g., prompts/reminders, more human involvement, training sessions or other co-interventions) and what impact the omission of these elements could have on use, adoption, or outcomes if the intervention is applied outside of a RCT setting.

	1	2	3	4	5	
subitem not at all important	○	○	●	○	○	essential

Clear selection

Does your paper address subitem 21-ii?

Copy and paste relevant sections from the manuscript (include quotes in quotation marks "like this" to indicate direct quotes from your manuscript), or elaborate on this item by providing additional information not in the ms, or briefly explain why the item is not applicable/relevant for your study

Yes: "
the MPS app was publicly available for download throughout the duration of our study, and participants were
not restricted to use other modalities or medications during this pilot which raises the possibility that treatment
effects might be cross-contaminated"

OTHER INFORMATION

23) Registration number and name of trial registry

Does your paper address CONSORT subitem 23? *

Copy and paste relevant sections from the manuscript (include quotes in quotation marks "like this" to indicate direct quotes from your manuscript), or elaborate on this item by providing additional information not in the ms, or briefly explain why the item is not applicable/relevant for your study

Yes: this can be accessed at ISRCTN18107122

24) Where the full trial protocol can be accessed, if available

Does your paper address CONSORT subitem 24? *

Cite a Multimedia Appendix, other reference, or copy and paste relevant sections from the manuscript (include quotes in quotation marks "like this" to indicate direct quotes from your manuscript), or elaborate on this item by providing additional information not in the ms, or briefly explain why the item is not applicable/relevant for your study

Yes: this can be accessed at ISRCTN18107122

25) Sources of funding and other support (such as supply of drugs), role of funders

Does your paper address CONSORT subitem 25? *

Copy and paste relevant sections from the manuscript (include quotes in quotation marks "like this" to indicate direct quotes from your manuscript), or elaborate on this item by providing additional information not in the ms, or briefly explain why the item is not applicable/relevant for your study

Yes: "Acknowledgements
Thank you to the Chief Science Office of Scotland for providing the funding for this research. Thank you
to MPS for being our technical partner in this project"

X27) Conflicts of Interest (not a CONSORT item)

X27-i) State the relation of the study team towards the system being evaluated

In addition to the usual declaration of interests (financial or otherwise), also state the relation of the study team towards the system being evaluated, i.e., state if the authors/evaluators are distinct from or identical with the developers/sponsors of the intervention.

	1	2	3	4	5	
subitem not at all important	◉	○	○	○	○	essential

Clear selection

Does your paper address subitem X27-i?

Copy and paste relevant sections from the manuscript (include quotes in quotation marks "like this" to indicate direct quotes from your manuscript), or elaborate on this item by providing additional information not in the ms, or briefly explain why the item is not applicable/relevant for your study

Not applicable to our pilot study

About the CONSORT EHEALTH checklist

As a result of using this checklist, did you make changes in your manuscript? *

- ○ yes, major changes
- ○ yes, minor changes
- ◉ no

What were the most important changes you made as a result of using this checklist?

Your answer

How much time did you spend on going through the checklist INCLUDING making changes in your manuscript *

3 hours

As a result of using this checklist, do you think your manuscript has improved? *

- (•) yes
- ( ) no
- ( ) Other:

Would you like to become involved in the CONSORT EHEALTH group?

This would involve for example becoming involved in participating in a workshop and writing an "Explanation and Elaboration" document

- (•) yes
- ( ) no
- ( ) Other:

Clear selection

Any other comments or questions on CONSORT EHEALTH

Your answer

STOP - Save this form as PDF before you click submit

To generate a record that you filled in this form, we recommend to generate a PDF of this page (on a Mac, simply select "print" and then select "print as PDF") before you submit it.

When you submit your (revised) paper to JMIR, please upload the PDF as supplementary file.

Don't worry if some text in the textboxes is cut off, as we still have the complete information in our database. Thank you!

Final step: Click submit !

Click submit so we have your answers in our database!

Submit

Clear form

Never submit passwords through Google Forms.

This content is neither created nor endorsed by Google. Report Abuse - Terms of Service - Privacy Policy

Google Forms
